# Genome-Wide Analysis of Attention Deficit Hyperactivity Disorder in Norway

**DOI:** 10.1371/journal.pone.0122501

**Published:** 2015-04-13

**Authors:** Tetyana Zayats, Lavinia Athanasiu, Ida Sonderby, Srdjan Djurovic, Lars T. Westlye, Christian K. Tamnes, Tormod Fladby, Heidi Aase, Pål Zeiner, Ted Reichborn-Kjennerud, Per M. Knappskog, Gun Peggy Knudsen, Ole A. Andreassen, Stefan Johansson, Jan Haavik

**Affiliations:** 1 K.G. Jebsen Centre for Neuropsychiatric Disorders, Department of Biomedicine, University of Bergen, Bergen, Norway; 2 NORMENT, K.G. Jebsen Centre for Psychosis Research, Division of Mental Health and Addiction, Oslo University Hospital & Institute of Clinical Medicine, University of Oslo, Oslo, Norway; 3 Department of Medical Genetics, Oslo University Hospital, Oslo, Norway; 4 NORMENT, K.G. Jebsen Centre for Psychosis Research, Department of Clinical Science, University of Bergen, Bergen, Norway; 5 Department of Psychology, University of Oslo, Oslo, Norway; 6 Research Group for Lifespan Changes in Brain and Cognition, Department of Psychology, University Of Oslo, Oslo, Norway; 7 Department of Neurology, Akershus University Hospital, Lørenskog, Norway; 8 Division of Mental Health, Norwegian Institute of Public Health, Oslo, Norway; 9 Oslo University Hospital, Child and Adolescent Mental Health Research Unit, Oslo, Norway; 10 University of Oslo, Institute of Clinical Medicine, Oslo, Norway; 11 K.G. Jebsen Centre for Neuropsychiatric Disorders, Department of Clinical Science, University of Bergen, Bergen, Norway; 12 Center for Medical Genetics and Molecular Medicine, Haukeland University Hospital, Bergen, Norway; 13 Division of Psychiatry, Haukeland University Hospital, Bergen, Norway; Kunming Institute of Zoology, Chinese Academy of Sciences, CHINA

## Abstract

**Background:**

Attention deficit hyperactivity disorder (ADHD) is a highly heritable neuropsychiatric condition, but it has been difficult to identify genes underlying this disorder. This study aimed to explore genetics of ADHD in an ethnically homogeneous Norwegian population by means of a genome-wide association (GWA) analysis followed by examination of candidate loci.

**Materials and Methods:**

Participants were recruited through Norwegian medical and birth registries as well as the general population. Presence of ADHD was defined according to DSM-IV criteria. Genotyping was performed using Illumina Human OmniExpress-12v1 microarrays. Statistical analyses were divided into several steps: (1) genome-wide association in the form of logistic regression in PLINK and follow-up pathway analyses performed in DAPPLE and INRICH softwares, (2) SNP-heritability calculated using genome-wide complex trait analysis (GCTA) tool, (3) gene-based association tests carried out in JAG software, and (4) evaluation of previously reported genome-wide signals and candidate genes of ADHD.

**Results:**

In total, 1.358 individuals (478 cases and 880 controls) and 598.384 autosomal SNPs were subjected to GWA analysis. No single polymorphism reached genome-wide significance. The strongest signal was observed at rs9949006 in the ENSG00000263745 gene (OR=1.51, 95% CI 1.28–1.79, p=1.38E-06). Pathway analyses of the top SNPs implicated genes involved in the regulation of gene expression, cell adhesion and inflammation. Among previously identified ADHD candidate genes, prominent association signals were observed for *SLC9A9* (rs1393072, OR=1.46, 95% CI = 1.21–1.77, p=9.95E-05) and *TPH2* (rs17110690, OR = 1.38, 95% CI = 1.14–1.66, p=8.31E-04).

**Conclusion:**

This study confirms the complexity and heterogeneity of ADHD etiology. Taken together with previous findings, our results point to a spectrum of biological mechanisms underlying the symptoms of ADHD, providing targets for further genetic exploration of this complex disorder.

## Introduction

Attention deficit hyperactivity disorder (ADHD) is one of the most common and most heritable childhood onset psychiatric conditions [1, 2]. Children with ADHD are at high risk of developing antisocial behavior, substance abuse and other psychiatric disorders, consequently presenting difficulties in their education and social integration [3]. Traditionally, ADHD was considered to be a childhood disorder that usually diminishes in adolescents. However, follow-up studies in the last few decades have clearly shown that many children continue to exhibit signs of ADHD in their adulthood as well [4, 5]. Persistence of ADHD poses a significant issue for society, with serious health-related, economic and personal consequences [6–9].

Despite the high heritability of 70–80% [1, 10, 11], the genetic architecture of ADHD is still largely unknown. So far, association studies of ADHD have implicated risk variants that (1) generally tend to have small effect sizes or be rare, (2) often refer to co-occurring conditions and (3) lack consistent replication [12, 13].

Neurotransmitters have been the major target for candidate gene association studies in ADHD. Nominal significance was reported for the dopamine-related genes SLC6A3 and DRD5; serotonin-related genes SLC6A4 and HTR1B; as well as a synaptic vesicle membrane docking SNAP-25 gene [14, 15]. However, effects of these genes are likely to be rather small and they have not been decisively supported by previous studies [16–19].

Genome-wide association (GWA) study is a useful tool for discovering novel risk variants as it allows a hypothesis-free interrogation of the entire genome. Several GWA analyses have been performed in order to identify ADHD risk loci using either case-control or family-based designs [13, 20], but to date there is no single nucleotide polymorphism (SNP) reaching the stringent genome-wide significance threshold (p<5.00E-08). Nonetheless, the top SNPs from previous GWA analyses include candidate genes that encode the cell adhesion protein CDH13 [16, 17, 21], the glutamate receptor GRM5 [22], the solute carrier protein SLC9A9 [23], the cholinergic receptor CHRNA7 [24] as well as the potassium-channel regulators KCNIP1, KCNIP4 and KCNC1 [16, 17].

The lack of robust genetic association findings in ADHD may be explained by its polygenic, multifactorial nature, with both common and rare variants likely contributing small effects to its etiology [24–26]. An additional potentially important factor may be the genetic heterogeneity of ADHD age-related subtypes (childhood versus adult ADHD) which may have different underlying genetic mechanisms. It is well established, for example, that age influences ADHD-relevant cognitive performance [27, 28]. In addition, it has been suggested that age can modulate the association of the *SLC6A3* gene with ADHD [29–31]. Nonetheless, persistent ADHD also has its onset in childhood and an overlap in genetics of childhood and adult ADHD may be observed from previous GWA studies. For example, *CDH13* encoding the cell adhesion protein T-cadherin is among the strongest associated candidate genes in both childhood and adult ADHD [16, 17]. Thus, performing GWA analysis on childhood and adult ADHD samples combined, as well as utilizing GWAS results in the examination of possibly involved biological processes, may help our understanding of genetic mechanisms underlying both childhood and adult ADHD.

This study aimed to identify genetic susceptibility loci of ADHD utilizing GWAS in a Norwegian sample of both childhood and adult ADHD, and investigate potential underlying mechanisms by pathway analyses.

## Materials and Methods

### Subjects

Recruitment was conducted at two sites in Norway: University of Bergen (UiB, Bergen, Norway) and the Norwegian Institute of Public Health (NIPH) in collaboration with the University of Oslo (UiO, Oslo, Norway). All participants provided signed informed consent form. The study was approved by the Norwegian regional medical research ethics committee West (IRB #3 FWA00009490, IRB00001872) as well as South East Norway, part C.

Recruitment of participants at UiB is described in details elsewhere [9]. In short, ADHD patients were recruited through a Norwegian national medical registry as well as by psychologists and psychiatrists working at out-patient clinics. ADHD diagnosis was defined according to DSM-IV criteria. Controls were randomly recruited through the Norwegian Medical Birth registry. All participants provided either blood or saliva samples for DNA extraction.

Participants at NIPH/UiO were selected through a screening procedure based on questionnaires from the Mother and Child Cohort Study (MoBa), resulting in 1195 children being clinically assessed [32]. The Norwegian Mother and Child Cohort Study (MoBa) is a prospective population-based pregnancy cohort study conducted by the Norwegian Institute of Public Health. Participants were recruited from all over Norway from 1999–2008 [33]. The Preschool Age Psychiatric Assessment [34] was used to determine symptoms of ADHD in accordance with DSM-IV criteria. Presence of significant symptoms of ADHD was defined as either 1) meeting all the symptom criteria for a DSM-IV-TR diagnosis, 2) meeting all the DSM-IV-TR symptom criteria for a diagnosis, but without report of impairment or 3) meeting at least three symptom criteria for a diagnosis in addition to report of impairment. DNA was available for 701 of the 1195 participants.

Additional control samples were recruited at UiO as parts of the following studies: Thematically Organized Psychosis Research (TOP) [35], LifeSpan Cognition and Plasticity through the Lifespan [36] and Neurocognitive Development [37], and Akershus University Hospital (AHUS) based memory study [38]. Healthy subjects in the TOP study were randomly selected using national records and the Primary Care Evaluation of Mental Disorders (PRIME-MD). None of the control subjects had a history of moderate/severe head injury, neurological disorder, mental retardation or an age outside the age range of 18–65 years. Subjects were excluded if they or any of their close relatives had a lifetime history of a severe psychiatric disorder (schizophrenia, bipolar disorder and major depression), a history of medical problems thought to interfere with brain function (hypothyroidism, uncontrolled hypertension and diabetes), or significant illicit drug use.

Participants from the Cognition and Plasticity through the Lifespan and Neurocognitive Development studies were recruited through newspaper advertisements, at local schools and among students and employees of the University of Oslo. The controls were screened for psychiatric disorders as well as neurological illnesses.

The AHUS sample consists of controls from longitudinal studies of age-related cognitive impairment. Any cognitive symptoms and somatic or psychiatric disease history with possible cognitive impact were among the exclusion criteria [38].

All individuals (cases and controls) recruited at UiB and within MoBa were screened for ADHD, while all other participants were screened for major neuropsychiatric disorders only (schizophrenia, bipolar disorder, major depression and mental retardation).

### Genotyping and quality control

Participants were genotyped on either Human OmniExpress-12v1-1_B (Illumina, San Diego, CA, USA) or Human OmniExpress-12v1_H (Illumina, San Diego, CA, USA) platforms. Genotyping was performed according to the standard Illumina protocol at Decode facility (Reykjavik, Iceland). Genotypes were assigned according to the standard Illumina protocol in GenomeStudio software, version V2011.1.

Individuals exhibiting high rates of genotype missingness (above 98%) or genome-wide heterozygosity (outside mean±3SD of the sample); cryptic relatedness (PI_HAT above 15%) or non-European ancestry were excluded from the analyses. Sex check was performed based on the homozygocity estimate of X chromosome markers implemented in PLINK. Given high concordance between the reported and estimated sex (>98% in our dataset), this method was also used to impute the missing sex information.

SNPs exhibiting high rates of missingness (above 95%), minor allele frequency (MAF) below 1% or failing Hardy-Weinberg equilibrium test (p<1.00E-05) were excluded from the analyses.

### Genome-wide association

Each SNP was tested for association with ADHD in the form of logistic regression assuming an underlying additive model and adjusted for gender as implemented in PLINK [39]. Because participants were genotyped on different arrays, SNPs showing high discrepancy in their frequencies between the two arrays (p<1.00E-05) were excluded from GWA analysis. A covariate corresponding to each genotyping array was included in the regression model when testing for association. Genomic control [40] was applied to check for possible population stratification. QQ plot was constructed to study the distribution of test statistics. A significance threshold of 5.00E-08 was adopted to correct for multiple testing.

### Expression Quantitative Trait Locus (eQTL) analysis

The top SNPs (p<1.00E-04) identified in genome-wide association tests were subjected to eQTL identification in Genevar software, using cis-eQTL SNP mode [41]. Expression-genotype pairs were extracted from HapMap3 data [42]. The reference source was set to Ensembl. The analyses were performed under default settings (Spearman’s correlation coefficient = 1, window around the SNPs of interest = 1 million basepairs, p-value threshold 1.00E-03).

### Estimation of SNP-heritability

SNP-heritability was estimated using the GCTA software [43]. Genetic similarity threshold was set to 0.05. The analysis model included sex and genotyping array as covariates.

### Enrichment analysis

To evaluate if any known biological pathways were implicated by our GWAS results, intervals around top SNPs (p<1.00E-04) were tested for enrichment in Gene Ontology (GO) nodes using the INRICH software [44]. Enrichment analysis performed in INRICH was based on the number of unique genes within an association interval that are over-represented in at least one defined gene-set. Association intervals were determined as the linkage-disequilibrium (LD) independent regions around the top associated SNPs. These regions were constructed by tagging the top SNPs in PLINK (tagging r^2^ threshold was set to 0.2, and each tags were constrained to be within a megabase). Defined gene-sets were determined as GO nodes. The minimum number of genes in a set was set to 5, while the maximum to 200 genes. Interval overlap was limited to 20 kbp up- or down-stream of a gene. Random interval sets, each approximately matching the associated intervals in terms of the number of SNPs and overlapping genes, were generated ten thousand times. To correct the empirical gene-set, p-value bootstrapping-based re-sampling (5,000 times) was applied.

### Protein-protein link evaluation

Using the same association intervals as determined in INRICH enrichment analysis, we assessed possible physical interactions between proteins encoded in those intervals. The analysis was performed using DAPPLE software [45]. DAPPLE identifies direct and indirect networks from proteins encoded in associated intervals by utilizing experimentally validated, protein-protein interaction databases. As a result, DAPPLE assesses if the connectivity between associated proteins would be greater than expected by chance.

### Gene-based association tests

Gene-based association tests were performed using JAG software [46, 47]. For each gene, the test statistic was defined as the sum of the—log_10_ association p-values of individual SNPs annotated to each of the genes. Gene annotation of the variants included a 2000 basepair region around each gene. Only genes with at least two annotated SNPs were considered for the analysis.

To ensure an unbiased interpretation of the results, 10.000 permutations were carried out. The statistics of each gene were computed for each permutation and the final gene-based p-value was calculated as the proportion of test statistics in the permuted data that was higher than the original test statistic. Genes reaching p-value below 1.00E-03 with the initial 10.000 permutations were further permuted 10 million times.

For permutations and to account for LD effects between examined SNPs, we utilized the genotype data of the European ancestry samples from the 1000 Genomes project [48].

### Analyses of previous ADHD GWA and candidate gene studies

After performing our analyses, we looked up previously published ADHD GWAS hits and SNPs in ADHD candidate genes in our results. Utilizing the catalogue of published GWAS (http://www.genome.gov/gwastudies/, December 2014), we curated a list of SNPs reaching p-value ≤ 1.00E-05 in previous genome-wide studies of ADHD. For ADHD candidate genes, we adopted the gene list constructed by Brookes et al [49]. These genes were annotated in our data with a 2.000 basepair window on each end of a gene.

### Meta-analysis of our main findings and PGC ADHD GWAS results

We have meta-analyzed our top hits (p-value < 1.00E-04) with the results of a large-scale ADHD meta-analysis completed by psychiatric genetics consortium (PGC) [50]. Meta-analysis were performed in the form of random effects regression implemented in PLINK.

## Results

### Genome-wide association

After quality control, there were 1.358 individuals (478 cases and 880 controls) and 598.467 SNPs available for the analysis. Details of the final sample are summarized in Table 1. Overall, the age distribution was comparable among the cases and controls (37.24% of the cases and 29.38% of the controls were children).

**Table 1:** Properties of the individuals subjected to GWAS in this study.

Recruiting center	Number of Cases	Number of Controls	Total number of participants	Mean Age (±SD)	Females (%)	Genotyping array
UiB	300	205	505	29.88 (9.14)	55.84	B
MoBa/Preschool ADHD (NIPH)	104	243	347	3.46 (0.12)	46.40	H
MoBa/Preschool ADHD (NIPH)	74	156	230	3.48 (0.11)	49.57	B
UiO controls	none	191	191	31.65 (18.09)	52.08	H
	none	85	85	65.22 (9.21)	54.02	B
Total	478	880	1358			

Genotyping array B refers to Human OmniExpress-12v1-1_B (Illumina, San Diego, CA, USA) and genotyping array H refers to Human OmniExpress-12v1_H (Illumina, San Diego, CA, USA). SD refers to standard deviation, UiB refers to University of Bergen and UiO refers to University of Oslo.

**Fig 1:**
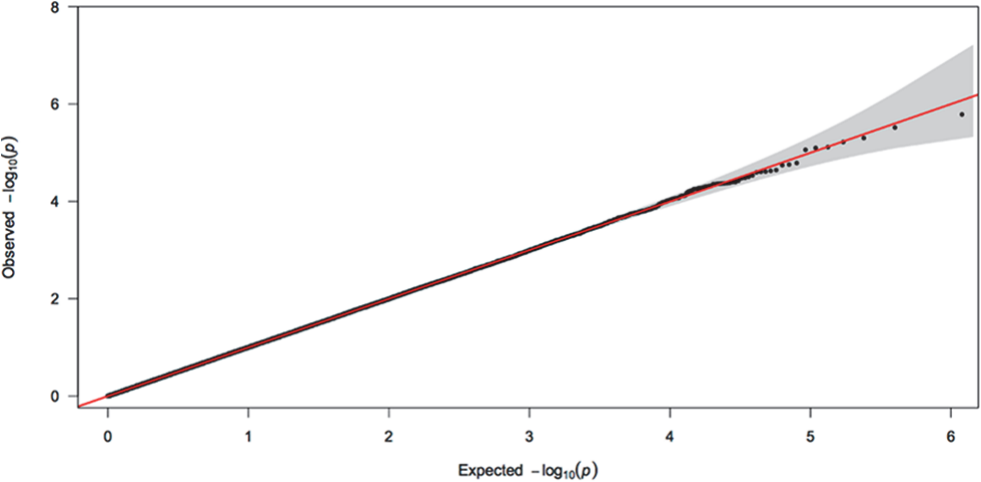
QQ plot. This figure represents the distribution of p-values observed in the presented genome-wide association study of ADHD. The shaded area represents the 95% confidence interval.

**Fig 2:**
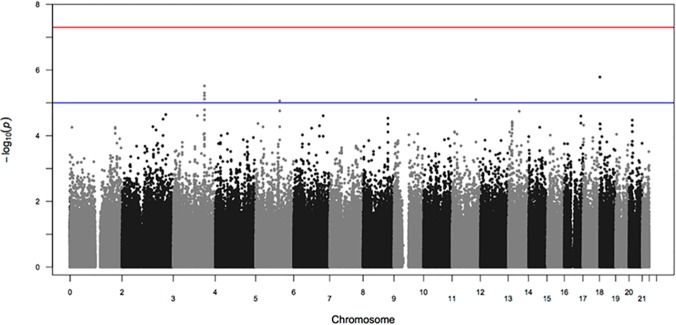
Manhattan plot. Red line represents genome-wide significance threshold of 5.00E-08, while the blue line corresponds to the suggestive threshold of p = 1.00E-05.

No variant reached genome-wide significance (p<5.00E-08). Table 2 details the top SNPs with association p-value being less than 1.00E-05 and S1A Table those reaching p-value below 1.00E-04. None of the main hits (p<1.00E-05) showed significant frequency difference between the two genotyping arrays utilized in this study (S3 Table). There was no inflation of calculated p-values observed (λ = 1.01). Figs 1 and 2 depict the QQ- and Manhattan-plots reflecting the results of the performed GWAS.

**Table 2:** List of SNPs with observed association p-value being less than 1.00E-05.

Chromosome	Basepair position	SNP	Annotation	reference allele	OR	95% CI	p-value
3	147951120	rs12497166	intergenic	T	0.68	0.58–0.80	4.95E-06
3	147967689	rs9836412	intergenic	A	0.68	0.57–0.80	4.18E-06
3	147978393	rs1019897	intergenic	C	0.67	0.57–0.79	2.55E-06
3	147986944	rs9834616	intergenic	A	0.68	0.58–0.81	6.25E-06
5	114497623	rs17137481	missense variant (N456S) in *TRIM36*	C	2.22	1.56–3.16	9.08E-06
11	113620851	rs2856244	Intronic variant in *ZBTB16*	A	1.47	1.24–1.75	8.69E-06
18	1906608	rs9949006	Long non-protein coding gene (ENSG00000263745)	T	1.51	1.28–1.79	1.38E-06

Chromosomal position is specified in Build 36 (hg18). OR refers to odds ratio and 95%CI refers to 95% confidence interval. *TRIM36* refers to tripartite motif containing 36 gene. *ZBTB16* refers to zinc finger and BTB domain containing 16 gene.

### Expression Quantitative Trait Locus (eQTL) analysis

We subjected our top seven SNPs detailed in Table 2 to eQTL evaluation in Genevar software. Matching transcripts were identified for two SNPs in an intergenic region on chromosome 3 (rs12497166 and rs1019897), rs17137481 in the *TRIM36* gene, rs9949006 in *ENSG00000263745* gene and rs2856244 in the vicinity of our top hit within *ZBTB16* gene. None of the probes revealed significant (p<1.00E-03) effects on any gene expression. S1 Fig summarizes the results of these analyses.

### Estimation of SNP-heritability

After removal of individuals showing genetic similarity over 0.05, 448 cases and 817 controls were analyzed. Overall, the SNP-heritability of ADHD was estimated to be 28% (standard error = 26%, p = 0.140).

### Enrichment analysis

There were 64 SNPs showing association of p<1.00E-04 and 45 LD-independent intervals were constructed (S1 Table). Out of these 45 intervals, 24 were intergenic and, thus, excluded from the analyses.

Overall, the associated intervals revealed enrichment in three GO pathways: rRNA processing (GO:0006364, p = 2.00E-03), skeletal system development (GO:0001501, p = 0.025) and central nervous system development (GO:0007417, p = 0.047). In particular, the enrichment was due to association endowment in the following genes: *UTP23*, *EXOSC8*, *ZBTB16*, *POSTN* and *ADAM23* (Table 3). Although none of these pathways reached significance after correcting for multiple testing, many implicate biological functions that are potentially relevant to ADHD.

**Table 3:** Results of enrichment analysis.

GO pathway	Empirical p-value	Corrected p-value	Associated Intervals	Gene list
GO:0006364 rRNA processing	0.002	0.50	chr8:117847902..117853398 chr13:36472896..36481557	UTP23, EXOSC8
GO:0001501 skeletal_system_development	0.025	0.94	chr11:113435620..113626627 chr13:37034758..37070894	ZBTB16, POSTN
GO:0007417 central_nervous_system_development	0.047	0.97	chr2:207016592..207190944 chr11:113435620..113626627	ADAM23,ZBTB16

This table details the GO pathways that revealed significant enrichment prior to correction for multiple testing.

### Protein-protein link evaluation

**Fig 3:**
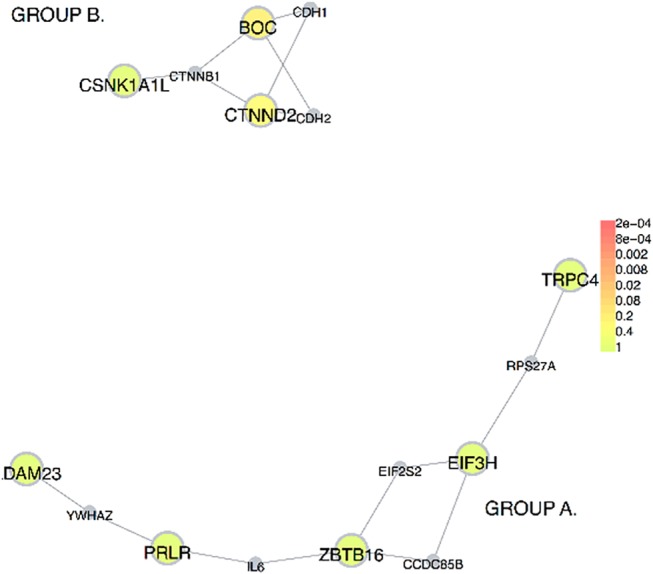
Protein-protein interaction network build from proteins encoded in associated intervals. The colored, full circles represent proteins encoded in associated intervals (S2 Table). The smaller, grey circles represent interactors of indirect connections. Functionally, the DAPPLE-constructed diagram can be divided into two main groups: group “A” mostly involved in the regulation of gene expression and inflammation; and group “B” mostly involved in cell adhesion.

The LD-independent associated intervals contained 28 genes (S2 Table) that were tested for protein-protein interaction in DAPPLE software. DAPPLE could not identify 3 genes: OR3A2, DYTN and LOC200726. Analysis of the remaining genes revealed no direct connections among proteins in our associated intervals. Nonetheless, several significant non-direct interactors were identified. This may suggest that although proteins encoded by genes in our associated intervals do not interact directly with each other, they may represent converging hubs of ADHD-relevant protein networks. Table 4 and Fig 3 present the details of DAPPLE results.

**Table 4:** Results of protein-protein link evaluation in DAPPLE. List of indirect interactors.

Protein	number of binding proteins	Binding proteins	crude p-value	corrected p-value	Function
CDH1	2	CTNND2, BOC	0.001	0.008	Calcium-dependent cell-adhesion protein
CDH2	2	CTNND2, BOC	0.001	0.008	Calcium-dependent cell-adhesion protein
IL6	2	PRLR, ZBTB16	0.001	0.008	Cytokine functioning in inflammation and the maturation of B cell
EIF2S2	2	EIF3H, ZBTB16	0.005	0.039	Eukaryotic translation initiation factor 2
CTNNB1	3	CTNND2, BOC, CSNK1A1L	0.005	0.039	Adherens junction protein, adhesion between cells

Presented p-values reflect the probability that by chance individual interactors would be as connected to seed proteins (S2 Table) as was observed in the constructed network.

### Gene-based association tests

In total, our dataset contained 16.546 genes with at least two annotated variants that were tested for gene-based association. Seventeen genes revealed p-values below 1.00E-03, with the most prominent signal observed for *CCRN4L* (p = 2.00E-07). We observed three SNPs annotated to *CCRN4L* that contributed to the detected gene-wide signal: rs10212985 (p = 1.48E-03), rs13108158 (p = 1.53E-03) and rs1112828 (p = 3.11E-04). S4 Table reports the details of the top hits in the gene-based analysis.

### Analyses of previous ADHD GWA and candidate gene studies

Based on the information of the catalogue of published GWAS studies (http://www.genome.gov/gwastudies/), we curated a list of 159 SNPs with reported p-value ≤ 1.00E-05 in previous GWA analyses of ADHD. Out of these 159 SNPs, only two revealed significant result with p-value below 0.05 in our analysis: rs2241685 and rs7463256 (p-value in our study is 4.76E-03 and 0.01 respectively). The first SNP is an intronic variant in the *MYT1L* gene found to be associated with adult ADHD (reported p = 8.00E-06), while the second SNP is an intronic one in the *CHMP7* gene and was noted in a meta-analysis of ADHD in children (reported p = 3.00E-06) [16, 21]. Since no odds ratio and standard error was reported, we were unable to meta-analyze our data with these previously published results. S5 Table contains details of all top hits (p-value ≤ 1.00E-06) from previous GWA analyses pursued in our study.

To analyze SNPs within previously reported ADHD candidate genes, we utilized the list of 51 such genes curated by Brookes et al [49]. Overall, our data contained 826 SNPs in these candidate genes and 16 of them revealed p-values below 0.01 in the following genes: *ADRA1A*, *DDC*, *PER2*, *SLC9A9*, *STX1A* and *TPH2* (S6 Table). *SLC9A9* revealed 7 significant SNPs with the strongest signal being rs1393072 (OR = 1.46, 95% CI = 1.21–1.77, p = 9.95E-05). *TPH2* was noted as the second most prominent gene with 5 significant SNPs and its strongest signal being rs17110690 (OR = 1.38, 95% CI = 1.14–1.66, p = 8.31E-04). Gene-based association tests affirmed these observations as only *SLC9A9* and *TPH2* genes reached overall p-values below 0.05 (p = 0.047 for *SLC9A9* based on 209 SNPs and p = 0.015 for *TPH2* based on 32 SNPs). S2 Fig depicts regional plots representing observed association signals annotated to *SLC9A9* and *TPH2* in this study.

### Meta-analysis of our main findings and PGC ADHD GWAS results

Apart from examining previously reported ADHD candidate genes and GWAS hits, we also performed a meta-analysis of our top SNPs (p<1.00E-04) with the data from a large-scale ADHD GWAS meta-analysis conducted by PGC. Out of the 64 most significant SNPs observed in our study (S1A Table), 47 were available in the PGC data. The strongest signal was observed for rs11121424 (p = 4.32E-05) in the LOC100506022 gene (S7 Table).

## Discussion

This is the first ADHD GWA analysis performed in the Norwegian population. Similarly to previous ADHD studies, we found no genome-wide significant SNPs at the standard genome-wide significance threshold (p<5.00E-08). However, several nominally significant (p<1.00E-05) variants were identified (Table 2). In addition, pathways analyses of associated intervals revealed a number of biological processes as well as protein interactions that are potentially relevant in the pathogenesis of ADHD (Tables 3 and 4).

The strongest signal in this GWAS was observed for rs9949006 on chromosome 18 (OR = 1.51, 95% CI 1.28–1.79, p = 1.64E-06). This SNP is a transcript variant of the non-coding RNA ENSG00000263745 gene. We have evaluated a possible function of rs9949006 using SNPinfo webserver (http://snpinfo.niehs.nih.gov), where no obvious gene-expression regulating activity was observed for this SNP. Nonetheless, non-protein coding RNAs play a critical role in regulation of gene expression and have been associated with a spectrum of human disorders, including neurodegeneration [51] and schizophrenia [52]. Non-coding RNA genes have also been observed among top hits in previous ADHD GWAS (S5 Table). In addition, it has been recently observed that SNPs previously associated with neurological and psychiatric conditions may be highly concentrated in the regions of long non-protein coding RNA genes [53].

Among our most significant SNPs, we have also noted a region on chromosome 3 as well as the *TRIM36* and *ZBTB16* genes (Table 2). The region on chromosome 3 can be identified as the regulatory ENSR00001484632 transcription factor binding feature, while both TRIM36 and ZBTB16 encode proteins that are expressed in the brain and are involved in the cell cycle regulation [54]. Functional evaluation of these SNPs in SNPinfo server (http://snpinfo.niehs.nih.gov) revealed possible gene-expression altering activity for rs17137481 only. This missense variant in the *TRIM36* gene is predicted to be benign by both PolyPhen and SIFT. However, this SNP (rs17137481) is in strong LD (r^2^ = 0.826 in CEU population) with rs4146835, predicted to be a transcription-binding site (SNPinfo server). In addition, rs17137481 is also in strong LD with rs3805596 and rs2974527 (r^2^ = 0.885 and 0.826 respectively in CEU population), which are located in 3’-UTR region of the *TRIM36* gene and are anticipated to be microRNA binding sites (SNPinfo server).

The TRIM36 protein is a multidomain E3 ubiquitin ligase that interacts with centromere protein-H and may be involved in differentiation and development during embryogenesis [54, 55]. This protein may be involved in protein–protein interactions [56], with a function in cell adhesion [57], the process implicated in the pathogenesis of ADHD by several previous studies [16, 17, 21, 58].

The variant in *ZBTB16* is an intronic SNP involved in nonsense mediated RNA decay. Similarly to TRIM26, ZBTB16 is involved in cell cycle regulation by encoding a transcriptional repressor that was identified in patients with acute promyelocytic leukemia [59], while mutations in mice have revealed that ZBTB16 also plays an important role in skeletal development and spermatogonial stem-cell maintenance [60, 61]. Deletions of the chromosomal region containing ZBTB16 are known to associate with mental retardation, skeletal defects and genital hypoplasia (OMIM # 612447) [62]. Interestingly, ZBTB16 is associated with ethanol preference in mice [63]. It is well established that human ADHD patients have an increased risk of alcohol dependence and substance abuse [9, 64].

Apart from being involved in cell cycle regulation, both TRIM36 and ZBTB16 are also among genes in the reactome pathways of Class I MHC mediated antigen processing & presentation and Immune System (REACT_75842.1 and REACT_75820.1). Class I MHC pathways may be involved in brain development [65]. In addition, several neuro-immunological hypotheses have been offered as a possible explanation for the development of neuro-psychiatric disorders [66–68], including ADHD [69]. It is also known that some immune conditions (e.g. asthma) often co-occur with ADHD [70].

Examining enrichment of associated intervals among GO nodes revealed possible engagement of mechanisms involved in rRNA processing as well as skeletal and central nervous system development in the pathogenesis of ADHD (Table 3). The strongest enrichment was observed for rRNA processing (p = 2.00E-03) due to association signals in the regions containing *UTP23* and *EXOSC8* genes. Both *UTP23* (encoding a small subunit processome component) and *EXOSC8* (encoding exosome component) are involved in multiple cellular RNA processing and degradation events. Enrichment for these genes may suggest that, similarly to other neuro-developmental condition, gene expression regulating components could be involved in the etiology of ADHD [52, 71]. This observation is also in line with our main finding being located within a long non-protein coding RNA gene.

Interestingly, the *ZBTB16* gene, where we noted some of our most prominent single point associations, was contained by the region contributing to the enrichment observed for the development of both skeletal and central nervous systems. In addition, signals in two other regions, encompassing *POSTN* and *ADAM23* genes, also conferred enrichment for these two nodes. *POSTN* encodes the extracellular matrix glycoprotein periostin that is found in blood and peripheral tissues, while *ADAM23* encodes a membrane-anchored protein (metalloprotease). Protein products of both of these genes are involved in cell adhesion, cell-cell and cell-matrix interactions, playing an important role in a variety of biological processes, including ADHD-relevant neurogenesis.

Since GO nodes are based on gene annotations only, we also conducted a protein-protein link exploration in DAPPLE software that utilizes experimental data. The results of this analysis did not show any direct interaction between proteins encoded by our nominally ADHD-associated loci. However, a number of significant intermediate interactors was recognized, with five of them surviving correction for multiple testing: CDH1 and CDH2, IL6, EIF2S2 and CTNNB1 (Table 4). Thus, it could be hypothesized that these genes highlight a protein network that may be impaired in ADHD. These protein-protein interactions may implicate two major networks (Fig 3): (1) cell adhesion (CDH1, CDH2, CTNNB1, CTNND2, BOC and CSNK1A1L genes); and (2) gene expression regulation and inflammation (ADAM23, YWHAZ, EIF2S2, IL6, EIF3H, ZBTB16, RPS27A, TRPC4, CCDC85B and PRLR genes). The above pathways are in line with previous findings showing that dysregulation during brain development (e.g. neurite outgrowth) may be important in the pathology of ADHD [13, 16, 25, 72].

Association with ADHD in this study was also examined in the form of gene-based tests. The most significant signal was noted for *CCRN4L* (p = 2.00E-07) that encodes a component of the circadian clock or downstream effector of clock function. In mammals, the circadian timing system controls many aspects of behavior and physiology, with its disruptions being implicated in major neuro-psychiatric disorders (including ADHD) at behavioral, endocrine and molecular levels [73–75].

To investigate the contribution of common SNPs to ADHD liability, we have estimated SNP-heritability using GCTA software. Similarly to previous observation in the large sample of European ancestry [76], our evaluation revealed the heritability of 28%. However, it is important to note that the large standard error in our estimations mirror the limited power to reliably determine the SNP-heritability.

The results of this study have been evaluated in the light of previously identified ADHD candidate genes and genome-wide association scans. While none of the previous GWAS hits replicated in our study (S5 Table), two candidate genes displayed several signals of association. *SLC9A9* showed the strongest evidence of association with an intronic rs1393072**,** p-value of 9.95E-05 (S6 Table and S2 Fig). *SLC9A9* encodes a sodium/hydrogen exchanger and may be of particular relevance to ADHD. This gene was found to be associated with a combined type of ADHD and it was noted among main signals in previous genome-wide linkage and association studies of ADHD [13, 49, 77]. Another candidate gene with a number of association signals observed in this study was *TPH2* gene (S6 Table and S2 Fig). It encodes the enzyme tryptophan hydroxylase 2 that initiates serotonin synthesis in the nervous system [78]. Similarly to *SLC9A9*, the association between ADHD and *TPH2* has previously been reported in numerous studies [19, 49, 79–81], although some negative results have also been reported [82].

This study should be viewed in the light of its limitations. There was no genome-wide significant observation for any SNP. One explanation for this could be that our study is of modest size (478 cases and 880 controls) and has examined common (MAF>1%) variants only. Thus, it has low power to detect common variants of small effect sizes.

Although assuming that performing GWAS on joined childhood and adult ADHD samples may improve our understanding of ADHD, it may also be a potential limitation. Thus, clinical heterogeneity may weaken the association signals [83]. This may occur, for example, due to the use of different assessment protocols; or due to the real genetic heterogeneity among different subtypes of ADHD [84]. It is currently unknown to which degree genetic and phenotypic heterogeneity impacts gene discovery in ADHD, and, in particular, how the genetics of ADHD change across the lifetime (from childhood to persistent ADHD).

In summary, we did not identify any gene loci reaching genome-wide significance, but found several promising candidates. Although replication in independent samples is warranted, these findings underline the genetic and phenotypic heterogeneity of ADHD. Taken together with previous findings, our results confirm the connection between biological processes important for brain development and ADHD, providing targets for further genetic exploration of this complex disorder.

## Supporting Information

S1 Table Details of SNPs associated at p<1.00E-04 level and corresponding LD-independent association intervals. A) Associated SNPs with p < 1.00E-04. SNPs with p-value below 1.00E-05 are highlighted in bold. B) Association Intervals based on the tagging of the SNPs in part A. of this table.
(DOCX)

S2 Table List of Genes located within the associated intervals. (DOCX)

S3 Table Results of the inter-array frequency difference test for the nominally significant SNPs. (DOCX)

S4 Table Details of the top hits of gene-based association tests. A) List of the genes reaching gene-based association p-value below 1.00E-03. "no.snps" refers to the number of SNPs annotated to the specified gene and tested as gene-based association. B) SNPs within CCRN4L gene.
(DOCX)

S5 Table Top hits (p-value ≤ 1.00E-05) from previous GWAS analyses and their details in our GWAS analyses. "NR" stands for "not reported", "NA" stands for "non-applicable" and "-" stands for no data in our dataset. SNPs reaching significance at 5% level in our GWAS analyses are highlighted in bold.
(DOCX)

S6 Table Most significant SNPs (p<0.01) in this study within 51 previously reported ADHD candidate genes^*^. The most significant SNP is highlighted in bold.
(DOCX)

S7 Table Meta-analysis of the top hits observed in this study (p<1.00E-04) and the PGC ADHD GWAS meta-analysis. "P(Fixed)", "OR(Fixed)" and "P(Random)","OR(Random)" refer to p-values and odds ratios under fixed and random effects modeling. "OR" refers to odds ratio, "SE" refers to standard error, "I" refers to I^2^ heterogeneity measure and "Q" refers to Cochran's Q heterogeneity measure.
(DOCX)

S1 Fig Summary of the eQTL analyses of the top SNPs (and those in their 1 MegaBasepair vicinity) observed in this study. Top SNPs were defined as variants reaching p-value below 1.00E-05 in the performed GWAS. The SNPs are detailed in Table 2 in the main text. Results are presented in the form of graphs detailing expression of the probes containing the SNP of interest across its genomic region. Y axis refers to –log_10_ of the expression p-value, X axis refers to chromosomal position in basepairs and each colored line refers to the examined HapMap3 population. *A) rs12497166 in intergenic region on chromosome 3*. *B) rs1019897 in intergenic region on chromosome 3*. *C) rs17137481 in TRIM36 gene*. *D) rs9949006 in ENSG00000263745 gene*. *E) rs2856244 in the vicinity of our top hit within ZBTB16 gene*.
(TIFF)

S2 Fig Regional plots representing observed association signals annotated to SLC9A9 and TPH2 in this study. A) SNPs observed around SLC9A9 gene. B) SNPs observed around TPH2 gene
(TIFF)
