# Supplementary material for: Genome-Wide Analysis of Attention Deficit Hyperactivity Disorder in Norway
Source: PLoS One. 2015 Apr 13;10(4):e0122501. doi: 10.1371/journal.pone.0122501 (PMC4395400; doi:10.1371/journal.pone.0122501)
Supplement: S1 Table — A) Associated SNPs with p < 1.00E-04. SNPs with p-value below 1.00E-05 are highlighted in bold. B) Association Intervals based on the tagging of the SNPs in part A. of this table. (DOCX) [file pone.0122501.s001.docx]

Table S1. Details of SNPs associated at p<1.00E-04 level and corresponding LD-independent association intervals

1. Associated SNPs with p < 1.00E-04

SNPs with p-value below 1.00E-05 are highlighted in bold.

| SNP | CHR | BP (hg18) | Risk Allele | OR | 95% CI | p-value |
| --- | --- | --- | --- | --- | --- | --- |
| rs11121424 | 1 | 9434156 | T | 0.66 | 0.54-0.81 | 6.98E-05 |
| rs10779265 | 1 | 214758625 | T | 0.70 | 0.59-0.83 | 5.39E-05 |
| rs945453 | 1 | 214759292 | A | 0.70 | 0.59-0.83 | 4.97E-05 |
| rs12027943 | 1 | 214760414 | T | 0.71 | 0.60-0.84 | 7.63E-05 |
| rs1437711 | 2 | 146233281 | A | 1.45 | 1.21-1.73 | 5.79E-05 |
| rs1710630 | 2 | 161484992 | C | 1.41 | 1.19-1.68 | 8.46E-05 |
| rs13385986 | 2 | 194439607 | C | 0.61 | 0.48-0.77 | 5.41E-05 |
| rs3770976 | 2 | 207126839 | A | 1.57 | 1.27-1.92 | 1.83E-05 |
| rs16860670 | 3 | 114424374 | A | 0.51 | 0.37-0.70 | 3.40E-05 |
| rs1393072 | 3 | 144503560 | T | 1.46 | 1.21-1.77 | 9.95E-05 |
| **rs12497166** | **3** | **147951120** | **T** | **0.68** | **0.57-0.80** | **4.95E-06** |
| **rs9836412** | **3** | **147967689** | **A** | **0.67** | **0.57-0.80** | **4.18E-06** |
| rs7629060 | 3 | 147970158 | C | 1.44 | 1.22-1.71 | 2.23E-05 |
| **rs1019897** | **3** | **147978393** | **C** | **0.67** | **0.57-0.79** | **2.55E-06** |
| **rs9834616** | **3** | **147986944** | **A** | **0.68** | **0.58-0.80** | **6.25E-06** |
| rs10049183 | 3 | 148022063 | G | 1.40 | 1.18-1.66 | 8.93E-05 |
| rs1608002 | 3 | 148038781 | G | 0.69 | 0.58-0.81 | 1.43E-05 |
| rs2013690 | 3 | 148045114 | C | 0.69 | 0.59-0.82 | 1.98E-05 |
| rs1978683 | 3 | 148053557 | G | 0.71 | 0.60-0.84 | 8.22E-05 |
| rs664330 | 4 | 27987893 | G | 0.68 | 0.57-0.82 | 6.90E-05 |
| rs9995833 | 4 | 57793006 | G | 0.64 | 0.51-0.80 | 9.06E-05 |
| rs1012176 | 5 | 11373538 | A | 0.66 | 0.54-0.81 | 6.36E-05 |
| rs6869985 | 5 | 35340938 | G | 1.73 | 1.32-2.26 | 6.30E-05 |
| rs2974520 | 5 | 114480533 | G | 2.17 | 1.49-3.15 | 5.05E-05 |
| **rs17137481** | **5** | **114497623** | **C** | **2.22** | **1.56-3.16** | **9.08E-06** |
| rs1422069 | 5 | 114498969 | A | 2.35 | 1.59-3.48 | 1.86E-05 |
| rs12190678 | 6 | 85091558 | A | 0.56 | 0.43-0.74 | 5.05E-05 |
| rs6420722 | 6 | 122295593 | G | 0.61 | 0.48-0.78 | 5.79E-05 |
| rs9321730 | 6 | 139944463 | A | 1.45 | 1.20-1.74 | 9.20E-05 |
| rs727098 | 6 | 139953015 | C | 1.50 | 1.24-1.80 | 2.13E-05 |
| rs6469653 | 8 | 117701145 | C | 0.68 | 0.57-0.81 | 1.97E-05 |
| rs10094824 | 8 | 117706044 | T | 0.68 | 0.56-0.82 | 6.41E-05 |
| rs11987235 | 8 | 117710791 | C | 0.64 | 0.52-0.79 | 3.89E-05 |
| rs6559453 | 9 | 71116478 | A | 0.61 | 0.47-0.78 | 8.69E-05 |
| rs7046956 | 9 | 112997355 | C | 1.45 | 1.20-1.74 | 7.98E-05 |
| rs7131034 | 11 | 12139612 | A | 0.66 | 0.54-0.81 | 9.50E-05 |
| rs2016398 | 11 | 12142377 | A | 0.64 | 0.51-0.80 | 8.75E-05 |
| rs7111329 | 11 | 23777209 | G | 1.87 | 1.38-2.55 | 5.77E-05 |
| **rs2856244** | **11** | **113620851** | **A** | **1.47** | **1.24-1.74** | **8.69E-06** |
| rs7317288 | 13 | 32902017 | T | 1.40 | 1.18-1.66 | 9.64E-05 |
| rs1924422 | 13 | 36438214 | G | 0.59 | 0.45-0.76 | 5.78E-05 |
| rs9547707 | 13 | 36439423 | C | 0.59 | 0.45-0.76 | 5.94E-05 |
| rs2323165 | 13 | 36467200 | T | 0.58 | 0.45-0.76 | 4.40E-05 |
| rs9547715 | 13 | 36472714 | G | 0.58 | 0.45-0.76 | 4.67E-05 |
| rs2147169 | 13 | 36483665 | C | 0.59 | 0.45-0.76 | 5.04E-05 |
| rs6563507 | 13 | 36488967 | T | 0.58 | 0.45-0.75 | 4.67E-05 |
| rs1199984 | 13 | 36543615 | G | 0.54 | 0.40-0.73 | 8.37E-05 |
| rs2399397 | 13 | 36641666 | T | 0.61 | 0.48-0.78 | 9.77E-05 |
| rs7324611 | 13 | 37011615 | A | 0.70 | 0.59-0.83 | 5.38E-05 |
| rs9315501 | 13 | 37012245 | C | 0.70 | 0.59-0.83 | 5.56E-05 |
| rs11843091 | 13 | 70503575 | A | 1.67 | 1.32-2.11 | 1.86E-05 |
| rs9550221 | 13 | 112525346 | T | 1.43 | 1.20-1.71 | 8.59E-05 |
| rs4903004 | 14 | 71792485 | C | 0.71 | 0.60-0.84 | 5.79E-05 |
| rs7201671 | 16 | 78953659 | G | 1.47 | 1.22-1.77 | 5.56E-05 |
| rs9940469 | 16 | 78956421 | A | 1.50 | 1.24-1.81 | 3.34E-05 |
| rs231674 | 17 | 3137283 | A | 1.42 | 1.20-1.67 | 4.27E-05 |
| rs920351 | 18 | 1862421 | A | 1.46 | 1.22-1.75 | 3.43E-05 |
| **rs9949006** | **18** | **1906608** | **T** | **1.52** | **1.28-1.80** | **1.38E-06** |
| rs10853291 | 18 | 3173354 | T | 0.71 | 0.60-0.84 | 7.91E-05 |
| rs2625379 | 18 | 11042389 | G | 1.40 | 1.18-1.65 | 8.24E-05 |
| rs3760948 | 19 | 4819802 | T | 0.70 | 0.58-0.83 | 7.59E-05 |
| rs6079838 | 20 | 15383756 | T | 1.44 | 1.21-1.72 | 4.06E-05 |
| rs6034195 | 20 | 15384903 | G | 1.43 | 1.20-1.70 | 5.87E-05 |
| rs6043561 | 20 | 15761644 | C | 0.70 | 0.58-0.83 | 7.47E-05 |

B) Association Intervals based on the tagging of the SNPs in part A. of this table

| CHR | START (hg18) | END (hg18) |
| --- | --- | --- |
| 1 | 214756955 | 214767865 |
| 1 | 9434156 | 9455825 |
| 2 | 146172331 | 146360816 |
| 2 | 161483735 | 161599628 |
| 2 | 193913619 | 194558196 |
| 2 | 207013899 | 207243698 |
| 3 | 114424247 | 114479831 |
| 3 | 144503560 | 144550940 |
| 3 | 147821338 | 148067154 |
| 3 | 147853723 | 148067154 |
| 3 | 147884884 | 148053557 |
| 3 | 147887741 | 148053557 |
| 3 | 147887741 | 148067154 |
| 4 | 27835432 | 28172553 |
| 4 | 57793006 | 57831125 |
| 5 | 11373538 | 11375037 |
| 5 | 114480533 | 114508263 |
| 5 | 35256622 | 35361312 |
| 6 | 122293921 | 122374297 |
| 6 | 139937839 | 140000063 |
| 6 | 85091558 | 85091558 |
| 8 | 117661610 | 117886437 |
| 8 | 117664045 | 117853156 |
| 8 | 117669148 | 117791736 |
| 9 | 112871140 | 113080147 |
| 9 | 71065278 | 71144434 |
| 11 | 113607375 | 113625984 |
| 11 | 12128635 | 12216931 |
| 11 | 12128635 | 12221659 |
| 11 | 23761408 | 23820976 |
| 13 | 112509005 | 112557920 |
| 13 | 32900488 | 32996232 |
| 13 | 36404819 | 36641666 |
| 13 | 36966250 | 37180470 |
| 13 | 70494388 | 70578522 |
| 14 | 71757027 | 71922573 |
| 16 | 78953465 | 78956421 |
| 17 | 3040628 | 3272631 |
| 18 | 11042389 | 11063732 |
| 18 | 1829387 | 1906608 |
| 18 | 1862421 | 1913315 |
| 18 | 3158816 | 3178976 |
| 19 | 4817451 | 4826765 |
| 20 | 15383203 | 15430941 |
| 20 | 15754820 | 15864192 |
