# Supplementary material for: Genome-Wide Analysis of Attention Deficit Hyperactivity Disorder in Norway
Source: PLoS One. 2015 Apr 13;10(4):e0122501. doi: 10.1371/journal.pone.0122501 (PMC4395400; doi:10.1371/journal.pone.0122501)
Supplement: S2 Table — (DOCX) [file pone.0122501.s002.docx]

Table S2. List of Genes located within the associated intervals.

| CHR | interval in basepairs (hg18) | Gene |
| --- | --- | --- |
| chr1 | 214756955..214767865 | ESRRG estrogen-related receptor gamma |
| chr2 | 207013899..207243698 | ADAM23 ADAM metallopeptidase domain 23 |
| chr2 | 207013899..207243698 | LOC200726 hCG1657980 |
| chr2 | 207013899..207243698 | DYTN dystrotelin |
| chr3 | 114424247..114479831 | BOC Boc homolog (mouse) |
| chr3 | 144503560..144550940 | SLC9A9 solute carrier family 9 (sodium/hydrogen exchanger), member 9 |
| chr5 | 11373538..11375037 | CTNND2 catenin (cadherin-associated protein), delta 2 (neural plakophilin-related arm-repeat protein) |
| chr5 | 35256622..35361312 | PRLR prolactin receptor |
| chr5 | 114480533..114508263 | TRIM36 tripartite motif-containing 36 |
| chr8 | 117661610..117886437 | EIF3H eukaryotic translation initiation factor 3, subunit H |
| chr8 | 117661610..117886437 | UTP23 small subunit (SSU) processome component, homolog (yeast) |
| chr11 | 12128635..12221659 | MICAL2 microtubule associated monoxygenase, calponin and LIM domain containing 2 |
| chr11 | 113607375..113625984 | ZBTB16 zinc finger and BTB domain containing 16 |
| chr13 | 36404819..36641666 | ALG5 asparagine-linked glycosylation 5, dolichyl-phosphate beta-glucosyltransferase homolog (S. cerevisiae) |
| chr13 | 36404819..36641666 | EXOSC8 exosome component 8 |
| chr13 | 36404819..36641666 | CSNK1A1L casein kinase 1, alpha 1-like |
| chr13 | 36966250..37180470 | POSTN periostin, osteoblast specific factor |
| chr13 | 36966250..37180470 | TRPC4 transient receptor potential cation channel, subfamily C, member 4 |
| chr13 | 112509005..112557920 | ATP11A ATPase, class VI, type 11A |
| chr14 | 71757027..71922573 | RGS6 regulator of G-protein signaling 6 |
| chr17 | 3040628..3272631 | OR1A2 olfactory receptor, family 1, subfamily A, member 2 |
| chr17 | 3040628..3272631 | OR1A1 olfactory receptor, family 1, subfamily A, member 1 |
| chr17 | 3040628..3272631 | OR3A2 olfactory receptor, family 3, subfamily A, member 2 |
| chr17 | 3040628..3272631 | OR3A1 olfactory receptor, family 3, subfamily A, member 1 |
| chr17 | 3040628..3272631 | OR1E1 olfactory receptor, family 1, subfamily E, member 1 |
| chr17 | 3040628..3272631 | OR3A3 olfactory receptor, family 3, subfamily A, member 3 |
| chr18 | 3158816..3178976 | MYOM1 myomesin 1, 185kDa |
| chr20 | 15383203..15430941 | MACROD2 MACRO domain containing 2 |
