# Supplementary material for: Genome-Wide Analysis of Attention Deficit Hyperactivity Disorder in Norway
Source: PLoS One. 2015 Apr 13;10(4):e0122501. doi: 10.1371/journal.pone.0122501 (PMC4395400; doi:10.1371/journal.pone.0122501)
Supplement: S3 Table — (DOCX) [file pone.0122501.s003.docx]

Table S3. Results of the inter-array frequency difference test for the nominally significant SNPs.

| Chromosome | Basepair position | SNP | Annotation | Risk allele | OR | 95% CI | ADHD association p-value | Between-array comparison p-value |
| --- | --- | --- | --- | --- | --- | --- | --- | --- |
| 3 | 147951120 | rs12497166 | intergenic | T | 0.68 | 0.58-0.80 | 6.04E-06 | 0.31 |
| 3 | 147967689 | rs9836412 | intergenic | A | 0.68 | 0.57-0.80 | 5.00E-06 | 0.39 |
| 3 | 147978393 | rs1019897 | intergenic | C | 0.67 | 0.57-0.79 | 3.04E-06 | 0.29 |
| 3 | 147986944 | rs9834616 | intergenic | A | 0.68 | 0.58-0.81 | 7.65E-06 | 0.39 |
| 5 | 114497623 | rs17137481 | *TRIM36* | C | 2.22 | 1.56-3.16 | 8.73E-06 | 0.84 |
| 11 | 113620851 | rs2856244 | *ZBTB16* | A | 1.47 | 1.24-1.75 | 7.99E-06 | 0.06 |
| 18 | 1906608 | rs9949006 | ENSG00000263745 | T | 1.51 | 1.28-1.79 | 1.64E-06 | 0.86 |
