# Supplementary material for: Genome-Wide Analysis of Attention Deficit Hyperactivity Disorder in Norway
Source: PLoS One. 2015 Apr 13;10(4):e0122501. doi: 10.1371/journal.pone.0122501 (PMC4395400; doi:10.1371/journal.pone.0122501)
Supplement: S4 Table — A) List of the genes reaching gene-based association p-value below 1.00E-03. "no.snps" refers to the number of SNPs annotated to the specified gene and tested as gene-based association. B) SNPs within CCRN4L gene. (DOCX) [file pone.0122501.s004.docx]

Table S4. Details of the top hits of gene-based association tests.

A) List of the genes reaching gene-based association p-value below 1.00E-03

“no.snps” refers to the number of SNPs annotated to the specified gene and tested as gene-based association.

| GENE | no.snps | p-value |
| --- | --- | --- |
| CCRN4L | 3 | 2.00E-07 |
| ALG5 | 6 | 3.45E-05 |
| EXOSC8 | 3 | 5.09E-05 |
| SUPT20H | 5 | 5.62E-05 |
| OR3A1 | 4 | 7.59E-05 |
| SAFB2 | 10 | 9.09E-05 |
| SHANK1 | 15 | 0.000156 |
| HOGA1 | 15 | 0.000236 |
| RPL13 | 4 | 0.000312 |
| ISM1 | 44 | 0.000325 |
| KLHL40 | 5 | 0.000339 |
| HHATL | 5 | 0.000339 |
| SUPT4H1 | 4 | 0.000391 |
| SH3BP5 | 31 | 0.000436 |
| POU4F1 | 3 | 0.000556 |
| TRIM36 | 14 | 0.000629 |
| C16orf58 | 8 | 0.000884 |

B) SNPs within CCRN4L gene

| SNP | CHR | BP (hg18) | Risk Allele | OR | 95% CI | p-value |
| --- | --- | --- | --- | --- | --- | --- |
| rs10212985 | 4 | 140172972 | T | 2.91 | 1.51 - 5.62 | 1.48E-03 |
| rs13108158 | 4 | 140175616 | A | 2.90 | 1.50 - 5.61 | 1.53E-03 |
| rs1112828 | 4 | 140176420 | A | 0.68 | 0.56 - 0.84 | 3.11E-04 |
