# Supplementary material for: Genome-Wide Analysis of Attention Deficit Hyperactivity Disorder in Norway
Source: PLoS One. 2015 Apr 13;10(4):e0122501. doi: 10.1371/journal.pone.0122501 (PMC4395400; doi:10.1371/journal.pone.0122501)
Supplement: S5 Table — "NR" stands for "not reported", "NA" stands for "non-applicable" and "-" stands for no data in our dataset. SNPs reaching significance at 5% level in our GWAS analyses are highlighted in bold. (DOCX) [file pone.0122501.s005.docx]

Table S5. Top hits (p-value ≤ 1.00E-05) from previous GWAS analyses and their details in our GWAS analyses.

“NR” stands for “not reported”, “NA” stands for “non-applicable” and “-“ stands for no data in our dataset. SNPs reaching significance at 5% level in our GWAS analyses are highlighted in bold.

| Previous GWAS | | | | | | This study | | | | | |
| --- | --- | --- | --- | --- | --- | --- | --- | --- | --- | --- | --- |
| PubmedID | SNP | Gene | Context | Risk Allele | p-value | OR | 95% CI | Risk Allele | OR | 95% CI | p-value |
| 22012869 | rs5016282 | GRM5 | intron | NR | 1.00E-06 | NR | NR | - | - | - | - |
| 22012869 | rs2556378 | BCL11A | intron | NR | 7.00E-06 | NR | NR | - | - | - | - |
| 22012869 | rs7984422 | CPG5 | intron | NR | 5.08E-06 | NR | NR | - | - | - | - |
| 22012869 | rs10838881 | intergenic | NA | NR | 9.29E-06 | NR | NR | - | - | - | - |
| 22012869 | rs2509689 | LOC102723838 | intron | NR | 5.62E-06 | NR | NR | T | 1.01 | 0.83 - 1.23 | 0.9152 |
| 18821565 | rs8047014 | HAS3 | NA | NR | 4.00E-06 | NR | NR | C | 1.13 | 0.96 - 1.33 | 0.1349 |
| 18821565 | rs2290416 | NAPRT1 | cds-synon | NR | 9.00E-06 | NR | NR | A | 0.83 | 0.62 - 1.12 | 0.229 |
| 18821565 | rs4147141 | Intergenic | NA | NR | 6.00E-06 | NR | NR | G | 1.1 | 0.93 - 1.31 | 0.2513 |
| 18821565 | rs1350666 | EREG | NA | NR | 8.00E-06 | NR | NR | A | 1.1 | 0.91 - 1.33 | 0.3056 |
| 18821565 | rs11719664 | ZNF385D | intron | NR | 2.00E-06 | NR | NR | A | 1.14 | 0.85 - 1.53 | 0.3813 |
| 18821565 | rs130575 | Intergenic | NA | NR | 5.00E-06 | NR | NR | C | 0.93 | 0.72 - 1.19 | 0.5476 |
| 18821565 | rs260461 | ZNF544 | intron | NR | 8.00E-06 | NR | NR | A | 1.07 | 0.85 - 1.33 | 0.5624 |
| 18821565 | rs7577925 | FLJ34870 | intron | NR | 3.00E-06 | NR | NR | T | 1.02 | 0.86 - 1.21 | 0.8432 |
| 18821565 | rs930421 | OXER1 | 3UTR | NR | 6.00E-06 | NR | NR | - | - | - | - |
| 18821565 | rs1514928 | Intergenic | NA | NR | 3.00E-06 | NR | NR | - | - | - | - |
| 18821565 | rs7992643 | CLYBL | intron | NR | 5.00E-06 | NR | NR | - | - | - | - |
| 18821565 | rs10767942 | Intergenic | NA | NR | 8.00E-06 | NR | NR | - | - | - | - |
| 18821565 | rs6791644 | FHIT | intron | NR | 8.00E-06 | NR | NR | - | - | - | - |
| 18821565 | rs1018040 | Intergenic | NA | NR | 5.00E-06 | NR | NR | - | - | - | - |
| 18821565 | rs1918172 | Intergenic | NA | NR | 5.00E-06 | NR | NR | - | - | - | - |
| 18821565 | rs17651978 | FOXP1 | intron | NR | 6.00E-06 | NR | NR | - | - | - | - |
| 18821565 | rs17367118 | Intergenic | NA | NR | 9.00E-06 | NR | NR | - | - | - | - |
| 18821565 | rs522958 | Intergenic | NA | NR | 1.00E-06 | NR | NR | - | - | - | - |
| 18821565 | rs272000 | Intergenic | NA | NR | 9.00E-06 | NR | NR | - | - | - | - |
| **18839057** | **rs2241685** | **MYT1L** | **intron** | **NR** | **8.00E-06** | **NR** | **NR** | **A** | **0.69** | **0.53 - 0.89** | **0.0047** |
| 18839057 | rs16928529 | UNC5B | intron | NR | 4.00E-06 | NR | NR | C | 1.13 | 0.95 - 1.35 | 0.1566 |
| 18839057 | rs2587695 | MGC33657 | intron | NR | 3.00E-07 | NR | NR | A | 1.1 | 0.94 - 1.29 | 0.2277 |
| 18839057 | rs2842643 | Intergenic | NA | NR | 3.00E-06 | NR | NR | G | 0.94 | 0.78 - 1.13 | 0.4904 |
| 18839057 | rs2199161 | MAP1B | intron | NR | 2.00E-06 | NR | NR | - | - | - | - |
| 18839057 | rs10786284 | TLL2 | intron | NR | 2.00E-06 | NR | NR | - | - | - | - |
| 18839057 | rs10983238 | ASTN2 | intron | NR | 1.00E-07 | NR | NR | - | - | - | - |
| 18839057 | rs11646411 | CDH13 | intron | NR | 7.00E-06 | NR | NR | - | - | - | - |
| 18839057 | rs412050 | PPM1F | nearGene-5 | NR | 6.00E-06 | NR | NR | - | - | - | - |
| 18839057 | rs2281597 | CSMD2 | intron | NR | 5.00E-07 | NR | NR | - | - | - | - |
| 18839057 | rs3799977 | SUPT3H | intron | NR | 5.00E-06 | NR | NR | - | - | - | - |
| 18839057 | rs469727 | REEP5 | intron | NR | 8.00E-06 | NR | NR | - | - | - | - |
| 18839057 | rs10514604 | ATP2C2 | intron | NR | 8.00E-07 | NR | NR | - | - | - | - |
| 18839057 | rs2242073 | CRYGC | intron | NR | 8.00E-06 | NR | NR | - | - | - | - |
| 18839057 | rs864643 | MOBP | intron | NR | 1.00E-08 | NR | NR | - | - | - | - |
| 18839057 | rs7995215 | GPC6 | intron | NR | 1.00E-08 | NR | NR | - | - | - | - |
| 18839057 | rs7175404 | intergenic | NA | NR | 6.00E-07 | NR | NR | - | - | - | - |
| 18839057 | rs2502731 | DNM1 | intron | NR | 2.00E-06 | NR | NR | - | - | - | - |
| 18839057 | rs1555322 | MMP24 | intron | NR | 4.00E-06 | NR | NR | - | - | - | - |
| 18839057 | rs4964805 | NT5DC3 | intron | NR | 5.00E-06 | NR | NR | - | - | - | - |
| 18839057 | rs2237349 | CREB5 | intron | NR | 5.00E-06 | NR | NR | - | - | - | - |
| 18839057 | rs2677744 | MAN2A2 | intron | NR | 1.00E-06 | NR | NR | - | - | - | - |
| 18839057 | rs515910 | C10orf79 | intron | NR | 4.00E-06 | NR | NR | - | - | - | - |
| 18839057 | rs220470 | ITGAE | intron | NR | 1.00E-07 | NR | NR | - | - | - | - |
| 18839057 | rs11243897 | C9orf98 | intron | NR | 6.00E-08 | NR | NR | - | - | - | - |
| 18839057 | rs7164335 | ITGA11 | intron | NR | 1.00E-07 | NR | NR | - | - | - | - |
| **20732625** | **rs7463256** | **CHMP7** | **intron** | **NR** | **3.00E-06** | **NR** | **NR** | **C** | **1.25** | **1.05 - 1.48** | **0.0104** |
| 20732625 | rs1464807 | Intergenic | NA | NR | 1.00E-06 | NR | NR | T | 1.11 | 0.88 - 1.39 | 0.3891 |
| 20732625 | rs12680109 | Intergenic | NA | NR | 7.00E-06 | NR | NR | C | 0.94 | 0.79 - 1.11 | 0.4518 |
| 20732625 | rs1027730 | Intergenic | NA | NR | 7.00E-06 | NR | NR | - | - | - | - |
| 20732625 | rs10485813 | Intergenic | NA | NR | 8.00E-06 | NR | NR | - | - | - | - |
| 20732626 | rs11074889 | EMP2 | intron | A | 7.00E-07 | 1.68 | NR | A | 0.82 | 0.64 - 1.05 | 0.1176 |
| 20732626 | rs2823819 | C21orf34 | intron | C | 7.00E-07 | 1.65 | NR | C | 1.19 | 0.95 - 1.49 | 0.1296 |
| 20732626 | rs1859156 | BMPR1B | intron | NR | 2.00E-06 | 1.54 | NR | T | 1.08 | 0.89 - 1.31 | 0.437 |
| 20732626 | rs10487524 | Intergenic | NA | T | 9.00E-06 | 1.6 | NR | T | 1.09 | 0.87 - 1.37 | 0.44 |
| 20732626 | rs10011926 | ELOVL6 | intron | NR | 8.00E-06 | 1.49 | NR | A | 0.95 | 0.78 - 1.16 | 0.6393 |
| 20732626 | rs9810857 | Intergenic | NA | T | 6.00E-06 | 1.41 | NR | T | 0.97 | 0.82 - 1.15 | 0.7434 |
| 20732626 | rs4923705 | Intergenic | NA | C | 2.00E-06 | 1.51 | NR | C | 0.99 | 0.82 - 1.20 | 0.9043 |
| 20732626 | rs438259 | Intergenic | NA | NR | 4.00E-06 | 1.49 | NR | - | - | - | - |
| 20732626 | rs2602381 | UGT1A9 | intron | A | 4.00E-06 | 1.42 | NR | - | - | - | - |
| 20732626 | rs8074751 | CCDC46 | intron | NR | 1.00E-06 | 1.45 | NR | - | - | - | - |
| 22420046 | rs11079828 | HOXB1 | nearGene-5 | T | 6.54E-06 | 1.32 | 1.17-1.49 | A | 1.01 | 0.86 - 1.20 | 0.8673 |
| 22420046 | rs3779312 | MAGI2 | intron | T | 8.38E-06 | 1.37 | 1.19-1.57 | T | 0.99 | 0.81 - 1.20 | 0.8955 |
| 22420046 | rs616668 | ATXN2 | intron | G | 8.62E-06 | 1.38 | 1.20-1.58 | - | - | - | - |
| 22420046 | rs42259 | TRIO | intron | T | 6.76E-06 | 1.41 | 1.22-1.64 | - | - | - | - |
| 22420046 | rs1744062 | NHEG1 | intron | G | 4.16E-06 | 0.75 | 0.67-0.85 | - | - | - | - |
| 23527680 | rs10521115 | Intergenic | NA | NR | 8.00E-06 | NR | NR | A | 1.14 | 0.95 - 1.37 | 0.1537 |
| 23527680 | rs11642377 | Intergenic | NA | NR | 7.00E-06 | NR | NR | A | 1.14 | 0.95 - 1.37 | 0.1572 |
| 23527680 | rs9364220 | Intergenic | NA | NR | 7.00E-06 | NR | NR | T | 1.14 | 0.95 - 1.36 | 0.1597 |
| 23527680 | rs9364220 | Intergenic | NA | NR | 6.00E-06 | NR | NR | T | 1.14 | 0.95 - 1.36 | 0.1597 |
| 23527680 | rs1822881 | Intergenic | NA | NR | 4.00E-06 | NR | NR | C | 1.08 | 0.90 - 1.29 | 0.4234 |
| 23527680 | rs4810796 | Intergenic | NA | NR | 3.00E-06 | NR | NR | A | 1.07 | 0.90 - 1.27 | 0.4449 |
| 23527680 | rs4810796 | Intergenic | NA | NR | 3.00E-06 | NR | NR | A | 1.07 | 0.90 - 1.27 | 0.4449 |
| 23527680 | rs4708431 | FRMD1 | nearGene-5 | NR | 7.00E-06 | NR | NR | C | 0.95 | 0.80 - 1.12 | 0.5184 |
| 23527680 | rs12931939 | GPR139 | intron | NR | 6.00E-06 | NR | NR | A | 0.94 | 0.78 - 1.13 | 0.5207 |
| 23527680 | rs12613775 | Intergenic | NA | NR | 4.00E-06 | NR | NR | T | 1.06 | 0.85 - 1.33 | 0.6072 |
| 23527680 | rs10180522 | Intergenic | NA | NR | 7.00E-06 | NR | NR | G | 1.05 | 0.87 - 1.26 | 0.619 |
| 23527680 | rs11681930 | PARD3B | intron | NR | 5.00E-06 | NR | NR | A | 1.04 | 0.85 - 1.27 | 0.6878 |
| 23527680 | rs2192271 | DPP6 | intron | NR | 1.00E-06 | NR | NR | A | 0.96 | 0.78 - 1.19 | 0.7147 |
| 23527680 | rs2119507 | Intergenic | NA | NR | 4.00E-06 | NR | NR | T | 1.04 | 0.81 - 1.33 | 0.7648 |
| 23527680 | rs12671878 | DPP6 | intron | NR | 1.00E-06 | NR | NR | T | 1.03 | 0.83 - 1.27 | 0.8127 |
| 23527680 | rs13006237 | Intergenic | NA | NR | 9.00E-06 | NR | NR | - | - | - | - |
| 23527680 | rs10153620 | PARD3B | intron | NR | 6.00E-06 | NR | NR | - | - | - | - |
| 23527680 | rs1036736 | Intergenic | NA | NR | 4.00E-06 | NR | NR | - | - | - | - |
| 23527680 | rs12513840 | LINC01183 | intron | NR | 7.00E-06 | NR | NR | - | - | - | - |
| 23527680 | rs7201408 | Intergenic | NA | NR | 7.00E-06 | NR | NR | - | - | - | - |
| 23527680 | rs4835929 | LINC01183 | intron | NR | 7.00E-06 | NR | NR | - | - | - | - |
| 23527680 | rs2608200 | Intergenic | NA | NR | 6.00E-06 | NR | NR | - | - | - | - |
| 23527680 | rs12919130 | Intergenic | NA | NR | 2.00E-06 | NR | NR | - | - | - | - |
| 23527680 | rs6119285 | DNMT3B | intron | NR | 4.00E-06 | NR | NR | - | - | - | - |
| 23527680 | rs10463832 | LINC01183 | intron | NR | 5.00E-06 | NR | NR | - | - | - | - |
| 23527680 | rs6758152 | Intergenic | NA | NR | 3.00E-06 | NR | NR | - | - | - | - |
| 23527680 | rs6057659 | Intergenic | NA | NR | 5.00E-06 | NR | NR | - | - | - | - |
| 23527680 | rs17123726 | Intergenic | NA | NR | 5.00E-06 | NR | NR | - | - | - | - |
| 23527680 | rs1515641 | LINC01183 | intron | NR | 4.00E-06 | NR | NR | - | - | - | - |
| 23527680 | rs6057648 | DNMT3B | intron | NR | 4.00E-06 | NR | NR | - | - | - | - |
| 23527680 | rs7270085 | MAPRE1 | 3UTR | NR | 4.00E-06 | NR | NR | - | - | - | - |
| 23527680 | rs12926725 | GPR139 | intron | NR | 3.00E-06 | NR | NR | - | - | - | - |
| 23527680 | rs11687420 | Intergenic | NA | NR | 4.00E-06 | NR | NR | - | - | - | - |
| 23527680 | rs6119286 | DNMT3B | intron | NR | 4.00E-06 | NR | NR | - | - | - | - |
| 23527680 | rs7448069 | CPLX2 | intron | NR | 1.00E-06 | NR | NR | - | - | - | - |
| 23527680 | rs2110267 | DPP6 | intron | NR | 6.00E-06 | NR | NR | - | - | - | - |
| 23527680 | rs6947495 | DPP6 | intron | NR | 1.00E-06 | NR | NR | - | - | - | - |
| 23527680 | rs2110267 | DPP6 | intron | NR | 5.00E-07 | NR | NR | - | - | - | - |
| 23527680 | rs12596252 | GPR139 | intron | NR | 3.00E-06 | NR | NR | - | - | - | - |
| 23527680 | rs1902813 | GPR139 | intron | NR | 3.00E-06 | NR | NR | - | - | - | - |
| 23527680 | rs910191 | intergenic | NA | NR | 2.00E-06 | NR | NR | - | - | - | - |
| 23527680 | rs11994034 | Intergenic | NA | NR | 7.00E-06 | NR | NR | - | - | - | - |
| 23527680 | rs4458264 | Intergenic | NA | NR | 3.00E-06 | NR | NR | - | - | - | - |
| 23527680 | rs4402823 | Intergenic | NA | NR | 3.00E-06 | NR | NR | - | - | - | - |
| 23527680 | rs13043694 | Intergenic | NA | NR | 3.00E-06 | NR | NR | - | - | - | - |
| 23527680 | rs8123073 | Intergenic | NA | NR | 5.00E-06 | NR | NR | - | - | - | - |
| 23527680 | rs6983777 | Intergenic | NA | NR | 8.00E-06 | NR | NR | - | - | - | - |
| 23527680 | rs910191 | Intergenic | NA | NR | 2.00E-06 | NR | NR | - | - | - | - |
| 23527680 | rs7164923 | Intergenic | NA | NR | 5.00E-06 | NR | NR | - | - | - | - |
| 23527680 | rs4458264 | Intergenic | NA | NR | 3.00E-06 | NR | NR | - | - | - | - |
| 23527680 | rs4402823 | Intergenic | NA | NR | 3.00E-06 | NR | NR | - | - | - | - |
| 23527680 | rs13043694 | Intergenic | NA | NR | 3.00E-06 | NR | NR | - | - | - | - |
| 23527680 | rs11903187 | Intergenic | NA | NR | 4.00E-06 | NR | NR | - | - | - | - |
| 23527680 | rs10193430 | Intergenic | NA | NR | 4.00E-06 | NR | NR | - | - | - | - |
| 23527680 | rs6497436 | Intergenic | NA | NR | 6.00E-06 | NR | NR | - | - | - | - |
| 23527680 | rs12926729 | Intergenic | NA | NR | 7.00E-06 | NR | NR | - | - | - | - |
| 23527680 | rs11647507 | Intergenic | NA | NR | 7.00E-06 | NR | NR | - | - | - | - |
| 23527680 | rs7185264 | Intergenic | NA | NR | 7.00E-06 | NR | NR | - | - | - | - |
| 23527680 | rs12924103 | Intergenic | NA | NR | 7.00E-06 | NR | NR | - | - | - | - |
| 23527680 | rs10521114 | Intergenic | NA | NR | 8.00E-06 | NR | NR | - | - | - | - |
| 23527680 | rs13407123 | Intergenic | NA | NR | 8.00E-06 | NR | NR | - | - | - | - |
| 23527680 | rs7722425 | LINC01183 | intron | NR | 5.00E-06 | NR | NR | - | - | - | - |
| 23527680 | rs10463833 | LINC01183 | intron | NR | 5.00E-06 | NR | NR | - | - | - | - |
| 23527680 | rs12523164 | LINC01183 | intron | NR | 6.00E-06 | NR | NR | - | - | - | - |
| 23527680 | rs6057651 | MAPRE1 | intron | NR | 4.00E-06 | NR | NR | - | - | - | - |
| 23527680 | rs6057652 | MAPRE1 | intron | NR | 4.00E-06 | NR | NR | - | - | - | - |
| 23527680 | rs6057652 | MAPRE1 | intron | NR | 7.00E-06 | NR | NR | - | - | - | - |
| 23527680 | rs6057651 | MAPRE1 | intron | NR | 7.00E-06 | NR | NR | - | - | - | - |
| 23728934 | rs6453417 | ARSB | intron | NR | 5.00E-06 | NR | NR | C | 0.92 | 0.79 - 1.08 | 0.3325 |
| 23728934 | rs4245040 | GRIK4 | intron | NR | 7.00E-06 | NR | NR | - | - | - | - |
| 23728934 | rs17232800 | Intergenic | NA | NR | 3.00E-06 | NR | NR | - | - | - | - |
| 23728934 | rs16828074 | Intergenic | NA | NR | 6.00E-09 | NR | NR | - | - | - | - |
| 25284319 | rs2415545 | intergenic | NA | A | 3.80E-06 | 1.47 | NA | A | 0.87 | 0.74 - 1.04 | 0.1207 |
| 25284319 | rs2415545 | intergenic | NA | A | 1.80E-06 | 1.59 | NA | A | 0.87 | 0.73 - 1.04 | 0.1207 |
| 25284319 | rs2415543 | intergenic | NA | A | 7.30E-06 | 1.55 | NA | A | 0.89 | 0.74 - 1.06 | 0.1968 |
| 25284319 | rs11646443 | KCNG4 | missense | G | 3.80E-07 | 1.61 | NA | A | 1.11 | 0.94 - 1.32 | 0.2179 |
| 25284319 | rs12590172 | FBXO33 | intron | NA | 2.80E-06 | NA | NA | G | 0.9 | 0.73 - 1.09 | 0.281 |
| 25284319 | rs3814860 | FBXO33 | 3UTR | A | 6.80E-06 | 1.61 | NA | T | 0.91 | 0.74 - 1.11 | 0.3375 |
| 25284319 | rs4416909 | intergenic | NA | C | 4.50E-06 | 1.54 | NA | T | 0.94 | 0.80 -1.11 | 0.4471 |
| 25284319 | rs1445594 | intergenic | NA | A | 3.70E-06 | 1.56 | NA | C | 0.94 | 0.79 - 1.12 | 0.4945 |
| 25284319 | rs17696574 | intergenic | NA | G | 2.50E-06 | 1.61 | NA | A | 0.95 | 0.80 - 1.12 | 0.5555 |
| 25284319 | rs11644983 | LOC102724084 | intron | C | 5.40E-06 | 2.08 | NA | A | 1.09 | 0.74 - 1.59 | 0.6729 |
| 25284319 | rs1988353 | ASIC2 | intron | A | 1.20E-06 | 1.88 | NA | A | 1.04 | 0.85 - 1.26 | 0.7134 |
| 25284319 | rs1897792 | intergenic | NA | A | 5.80E-06 | 1.56 | NA | A | 0.98 | 0.79 - 1.21 | 0.8314 |
| 25284319 | rs4902569 | intergenic | NA | G | 6.20E-06 | 1.5 | NA | - | - | - | - |
| 25284319 | rs4902569 | intergenic | NA | G | 5.80E-06 | 1.6 | NA | - | - | - | - |
| 25284319 | rs2038278 | FBXO33 | intron | NA | 7.70E-06 | NA | NA | - | - | - | - |
| 25284319 | rs10594 | PEX19 | 3UTR | G | 2.30E-06 | 1.49 | NA | - | - | - | - |
| 25284319 | rs2232429 | ZSCAN12 | intron | G | 7.70E-08 | 2.6 | NA | - | - | - | - |
