# Supplementary material for: Genome-Wide Analysis of Attention Deficit Hyperactivity Disorder in Norway
Source: PLoS One. 2015 Apr 13;10(4):e0122501. doi: 10.1371/journal.pone.0122501 (PMC4395400; doi:10.1371/journal.pone.0122501)
Supplement: S6 Table — The most significant SNP is highlighted in bold. (DOCX) [file pone.0122501.s006.docx]

Table S6. Most significant SNPs (p<0.01) in this study within 51 previously reported ADHD candidate genes^*^.

The most significant SNP is highlighted in bold.

| SNP | GENE | CHR | BP (hg18) | Risk Allele | OR | 95% CI | p-value |
| --- | --- | --- | --- | --- | --- | --- | --- |
| rs13278849 | ADRA1A | 8 | 26770791 | G | 1.44 | 1.19-1.73 | 1.75E-04 |
| rs11575544 | DDC | 7 | 50498256 | T | 0.60 | 0.41 - 0.87 | 7.22E-03 |
| rs10462023 | PER2 | 2 | 238849320 | A | 1.31 | 1.09 - 1.55 | 2.60E-03 |
| **rs1393072** | **SLC9A9** | **3** | **144503560** | **T** | **1.46** | **1.21 - 1.77** | **9.95E-05** |
| rs838610 | SLC9A9 | 3 | 144661216 | T | 1.28 | 1.08 - 1.52 | 4.62E-03 |
| rs13058809 | SLC9A9 | 3 | 144920814 | C | 1.41 | 1.11 - 1.80 | 5.19E-03 |
| rs2049504 | SLC9A9 | 3 | 144720109 | T | 0.71 | 0.55 - 0.91 | 7.91E-03 |
| rs2119380 | SLC9A9 | 3 | 144720343 | C | 0.79 | 0.67 - 0.94 | 8.73E-03 |
| rs7642361 | SLC9A9 | 3 | 144543077 | A | 0.76 | 0.61 - 0.93 | 9.03E-03 |
| rs7431684 | SLC9A9 | 3 | 144511780 | C | 1.25 | 1.05 - 1.48 | 9.76E-03 |
| rs6956879 | STX1A | 7 | 72751419 | A | 1.23 | 1.05 - 1.45 | 9.65E-03 |
| rs17110690 | TPH2 | 12 | 70694264 | T | 1.38 | 1.14 - 1.66 | 8.31E-04 |
| rs9325202 | TPH2 | 12 | 70693744 | T | 1.32 | 1.12 - 1.56 | 1.14E-03 |
| rs11179039 | TPH2 | 12 | 70688453 | A | 1.29 | 1.09 - 1.52 | 3.17E-03 |
| rs17110489 | TPH2 | 12 | 70634273 | G | 1.29 | 1.08 - 1.56 | 6.16E-03 |
| rs7963720 | TPH2 | 12 | 70652453 | G | 1.25 | 1.06 - 1.48 | 9.18E-03 |

^*^  as reported in Brookes, K., et al., *The analysis of 51 genes in DSM-IV combined type attention deficit hyperactivity disorder: association signals in DRD4, DAT1 and 16 other genes.* Mol Psychiatry, 2006. **11**(10): p. 934-53.
