# Supplementary material for: Genome-Wide Analysis of Attention Deficit Hyperactivity Disorder in Norway
Source: PLoS One. 2015 Apr 13;10(4):e0122501. doi: 10.1371/journal.pone.0122501 (PMC4395400; doi:10.1371/journal.pone.0122501)
Supplement: S7 Table — "P(Fixed)", "OR(Fixed)" and "P(Random)","OR(Random)" refer to p-values and odds ratios under fixed and random effects modeling. "OR" refers to odds ratio, "SE" refers to standard error, "I" refers to I2 heterogeneity measure and "Q" refers to Cochran's Q heterogeneity measure. (DOCX) [file pone.0122501.s007.docx]

Table S7. Meta-analysis of the top hits observed in this study (p<1.00E-04) and the PGC ADHD GWAS meta-analysis

“P(Fixed)”, “OR(Fixed)” and “P(Random)”,”OR(Random)” refer to p-values and odds ratios under fixed and random effects modeling. “OR” refers to odds ratio, “SE” refers to standard error, “I” refers to I^2^ heterogeneity measure and “Q” refers to Cochran’s Q heterogeneity measure.

|  | | | | Meta-Analysis | | | | | | Norwegian GWAS | | | PGC GWAS | | |
| --- | --- | --- | --- | --- | --- | --- | --- | --- | --- | --- | --- | --- | --- | --- | --- |
| CHR | BP | SNP | Risk Allele | P (Fixed) | P(Random) | OR(Fixed) | OR(Random) | Q | I | OR | SE | P | OR | SE | P |
| 1 | 9434156 | rs11121424 | T | 4.32E-05 | 0.05511 | 0.8153 | 0.7674 | 0.0188 | 81.88 | 0.6591 | 0.1034 | 5.55E-05 | 0.8698 | 0.057 | 0.0143 |
| 1 | 214758625 | rs10779265 | T | 0.1075 | 0.3697 | 0.9382 | 0.8511 | 0.0002 | 92.63 | 0.7054 | 0.08696 | 6.01E-05 | 1.011 | 0.0445 | 0.806 |
| 1 | 214759292 | rs945453 | T | 0.09399 | 0.3638 | 0.9353 | 0.8495 | 0.0002 | 92.58 | 0.7043 | 0.08699 | 5.58E-05 | 1.009 | 0.045 | 0.8417 |
| 2 | 146233281 | rs1437711 | A | 0.08132 | 0.3329 | 1.0685 | 1.1947 | 0.0003 | 92.5 | 1.449 | 0.09169 | 5.31E-05 | 1.003 | 0.0418 | 0.9425 |
| 2 | 161484992 | rs1710630 | A | 0.002757 | 0.1839 | 0.8866 | 0.8205 | 0.0026 | 88.98 | 0.7 | 0.08814 | 6.71E-05 | 0.9434 | 0.0452 | 0.1977 |
| 2 | 194439607 | rs13385986 | A | 0.01114 | 0.2522 | 1.1553 | 1.3022 | 0.0008 | 91.02 | 1.66 | 0.1226 | 3.12E-05 | 1.046 | 0.0642 | 0.4815 |
| 3 | 114424374 | rs16860670 | T | 0.07552 | 0.3596 | 0.8759 | 0.7262 | 0.0001 | 93.2 | 0.5051 | 0.1618 | 2.44E-05 | 1.016 | 0.084 | 0.8511 |
| 3 | 147951120 | rs12497166 | A | 0.1386 | 0.3806 | 0.9486 | 0.8383 | 0 | 94.59 | 0.6806 | 0.08504 | 6.04E-06 | 1.018 | 0.0392 | 0.647 |
| 3 | 147978393 | rs1019897 | A | 0.1457 | 0.3872 | 1.0532 | 1.1993 | 0 | 95.03 | 1.49 | 0.08515 | 3.04E-06 | 0.9785 | 0.0392 | 0.5788 |
| 3 | 148053557 | rs1978683 | T | 0.0711 | 0.327 | 1.0681 | 1.1803 | 0.0004 | 92.12 | 1.41 | 0.08607 | 8.60E-05 | 1.005 | 0.0403 | 0.9086 |
| 4 | 27987893 | rs664330 | A | 0.02436 | 0.2631 | 1.0913 | 1.2098 | 0.0011 | 90.68 | 1.45 | 0.09505 | 9.87E-05 | 1.031 | 0.0425 | 0.4761 |
| 4 | 57793006 | rs9995833 | T | 0.231 | 0.38 | 1.0539 | 1.2244 | 0.0002 | 92.95 | 1.56 | 0.113 | 8.62E-05 | 0.9831 | 0.0476 | 0.7212 |
| 5 | 11373538 | rs1012176 | T | 0.6299 | 0.4743 | 0.9798 | 0.841 | 0 | 94.51 | 0.6542 | 0.1037 | 4.27E-05 | 1.062 | 0.0463 | 0.192 |
| 5 | 35340938 | rs6869985 | G | 0.005721 | 0.2116 | 1.1706 | 1.3478 | 0.0015 | 90.11 | 1.739 | 0.1369 | 5.36E-05 | 0.9282 | 0.0627 | 0.2346 |
| 5 | 114480533 | rs2974520 | T | 0.1005 | 0.3795 | 0.8623 | 0.7007 | 0.0002 | 92.87 | 0.46 | 0.1905 | 5.32E-05 | 1.034 | 0.1024 | 0.7465 |
| 5 | 114497623 | rs17137481 | C | 0.02808 | 0.3137 | 1.1964 | 1.4816 | 0.0001 | 93.33 | 2.223 | 0.1796 | 8.73E-06 | 0.9823 | 0.0917 | 0.8449 |
| 5 | 114498969 | rs1422069 | A | 0.03687 | 0.3229 | 1.2122 | 1.5172 | 0.0002 | 92.9 | 2.354 | 0.1994 | 1.76E-05 | 1.012 | 0.104 | 0.9093 |
| 6 | 41690442 | rs2765943 | A | 0.09456 | 0.3503 | 1.1017 | 1.2706 | 0.0004 | 91.98 | 1.663 | 0.1302 | 9.46E-05 | 0.9952 | 0.0647 | 0.9408 |
| 6 | 85091558 | rs12190678 | T | 0.003278 | 0.1903 | 0.8112 | 0.7307 | 0.0033 | 88.38 | 0.567 | 0.1413 | 5.89E-05 | 0.9162 | 0.0824 | 0.2884 |
| 6 | 139953015 | rs727098 | C | 0.1654 | 0.3885 | 1.0579 | 1.1985 | 0.0001 | 93.77 | 1.491 | 0.09475 | 2.47E-05 | 1.021 | 0.0449 | 0.6452 |
| 8 | 117701145 | rs6469653 | A | 0.04708 | 0.3106 | 1.0757 | 1.2004 | 0.0002 | 92.71 | 1.45 | 0.08862 | 2.93E-05 | 1.011 | 0.0404 | 0.7828 |
| 8 | 117710791 | rs11987235 | A | 0.06222 | 0.3204 | 1.0872 | 1.2334 | 0.0003 | 92.27 | 1.54 | 0.1067 | 4.44E-05 | 1.009 | 0.0494 | 0.8501 |
| 9 | 71116478 | rs6559453 | T | 0.3652 | 0.4101 | 0.9568 | 0.8043 | 0.0001 | 93.28 | 0.6095 | 0.1267 | 9.33E-05 | 1.035 | 0.0529 | 0.5111 |
| 9 | 112997355 | rs7046956 | C | 0.7391 | 0.503 | 1.0132 | 1.1544 | 0 | 94.25 | 1.442 | 0.09328 | 8.78E-05 | 1.065 | 0.0433 | 0.1478 |
| 11 | 12139612 | rs7131034 | A | 0.8737 | 0.5559 | 1.0071 | 0.8585 | 0 | 94.91 | 0.6569 | 0.1062 | 7.60E-05 | 1.103 | 0.049 | 0.04541 |
| 11 | 23777209 | rs7111329 | G | 0.02996 | 0.2893 | 1.1704 | 1.358 | 0.001 | 90.75 | 1.841 | 0.1557 | 8.93E-05 | 0.9685 | 0.0819 | 0.6957 |
| 11 | 113620851 | rs2856244 | A | 0.1282 | 0.393 | 1.0608 | 1.1915 | 0 | 94.42 | 1.473 | 0.08672 | 7.99E-06 | 0.9771 | 0.0434 | 0.593 |
| 13 | 25009534 | rs951132 | C | 0.7579 | 0.4986 | 0.9885 | 0.868 | 0 | 94.33 | 0.6986 | 0.09076 | 7.73E-05 | 0.9418 | 0.0412 | 0.1448 |
| 13 | 36438214 | rs1924422 | G | 0.02809 | 0.2797 | 0.8868 | 0.7641 | 0.0006 | 91.62 | 0.5874 | 0.1312 | 4.98E-05 | 1.034 | 0.0602 | 0.5782 |
| 13 | 36439423 | rs9547707 | T | 0.02875 | 0.2797 | 1.1271 | 1.3078 | 0.0006 | 91.55 | 1.7 | 0.1314 | 5.15E-05 | 1.034 | 0.0602 | 0.5794 |
| 13 | 36467200 | rs2323165 | A | 0.03049 | 0.2862 | 0.8891 | 0.7619 | 0.0004 | 92.04 | 0.5823 | 0.1312 | 3.76E-05 | 0.9705 | 0.0597 | 0.617 |
| 13 | 36472714 | rs9547715 | T | 0.02624 | 0.28 | 1.1282 | 1.3158 | 0.0004 | 91.99 | 1.72 | 0.1311 | 4.00E-05 | 1.034 | 0.0596 | 0.5761 |
| 13 | 36483665 | rs2147169 | C | 0.02856 | 0.2816 | 0.888 | 0.7627 | 0.0005 | 91.84 | 0.5848 | 0.1311 | 4.29E-05 | 1.033 | 0.0596 | 0.5901 |
| 13 | 36488967 | rs6563507 | A | 0.02856 | 0.2825 | 0.888 | 0.7619 | 0.0004 | 91.92 | 0.5835 | 0.1311 | 4.00E-05 | 0.9685 | 0.0596 | 0.5919 |
| 13 | 36543615 | rs1199984 | G | 0.2685 | 0.3862 | 0.9361 | 0.757 | 0.0001 | 93.07 | 0.5402 | 0.1566 | 8.39E-05 | 0.9729 | 0.0646 | 0.6708 |
| 13 | 36641666 | rs2399397 | A | 0.2734 | 0.388 | 0.9492 | 0.7974 | 0.0001 | 93.34 | 0.6057 | 0.1253 | 6.34E-05 | 1.024 | 0.0515 | 0.6478 |
| 13 | 37012245 | rs9315501 | C | 0.01189 | 0.248 | 0.9077 | 0.8311 | 0.0009 | 90.91 | 0.7018 | 0.08659 | 4.33E-05 | 1.034 | 0.043 | 0.4335 |
| 13 | 70503575 | rs11843091 | A | 0.003179 | 0.2209 | 1.2037 | 1.3185 | 0.0013 | 90.35 | 1.669 | 0.1194 | 1.81E-05 | 1.062 | 0.0739 | 0.4131 |
| 15 | 33995600 | rs1497815 | A | 0.1427 | 0.3613 | 0.9498 | 0.8527 | 0.0002 | 92.75 | 0.71 | 0.08589 | 9.65E-05 | 1.007 | 0.0385 | 0.8635 |
| 17 | 3137283 | rs231674 | T | 0.001205 | 0.1578 | 1.1256 | 1.2159 | 0.0031 | 88.55 | 1.411 | 0.08476 | 4.79E-05 | 1.069 | 0.0405 | 0.09942 |
| 18 | 1862421 | rs920351 | A | 0.5233 | 0.4673 | 1.0248 | 1.1664 | 0 | 94.35 | 1.453 | 0.09142 | 4.35E-05 | 0.951 | 0.0423 | 0.2354 |
| 18 | 3161224 | rs7235847 | A | 0.1009 | 0.3479 | 1.0648 | 1.1879 | 0.0002 | 92.59 | 1.44 | 0.09062 | 6.20E-05 | 0.9973 | 0.0422 | 0.9499 |
| 18 | 3173354 | rs10853291 | T | 0.04225 | 0.3118 | 0.9245 | 0.8408 | 0.0004 | 92.07 | 0.7025 | 0.08647 | 4.43E-05 | 0.9901 | 0.0432 | 0.8194 |
| 19 | 4819802 | rs3760948 | T | 0.7479 | 0.528 | 0.9871 | 0.8743 | 0 | 94.36 | 0.7015 | 0.09056 | 9.06E-05 | 1.074 | 0.045 | 0.1144 |
| 20 | 15383756 | rs6079838 | A | 0.1265 | 0.3591 | 1.0575 | 1.1892 | 0.0001 | 93.29 | 1.449 | 0.08942 | 3.35E-05 | 0.9926 | 0.0401 | 0.8542 |
| 20 | 15384903 | rs6034195 | G | 0.2055 | 0.3884 | 1.0471 | 1.1763 | 0.0001 | 93.39 | 1.432 | 0.08833 | 4.84E-05 | 1.018 | 0.0399 | 0.6464 |
| 20 | 15761644 | rs6043561 | C | 0.05344 | 0.3166 | 0.9252 | 0.8389 | 0.0005 | 91.66 | 0.6976 | 0.09096 | 7.51E-05 | 1.009 | 0.0449 | 0.8399 |
