# Supplementary figures and images for: Genome-Wide Analysis of Attention Deficit Hyperactivity Disorder in Norway
Source: PLoS One. 2015 Apr 13;10(4):e0122501. doi: 10.1371/journal.pone.0122501 (PMC4395400; doi:10.1371/journal.pone.0122501)

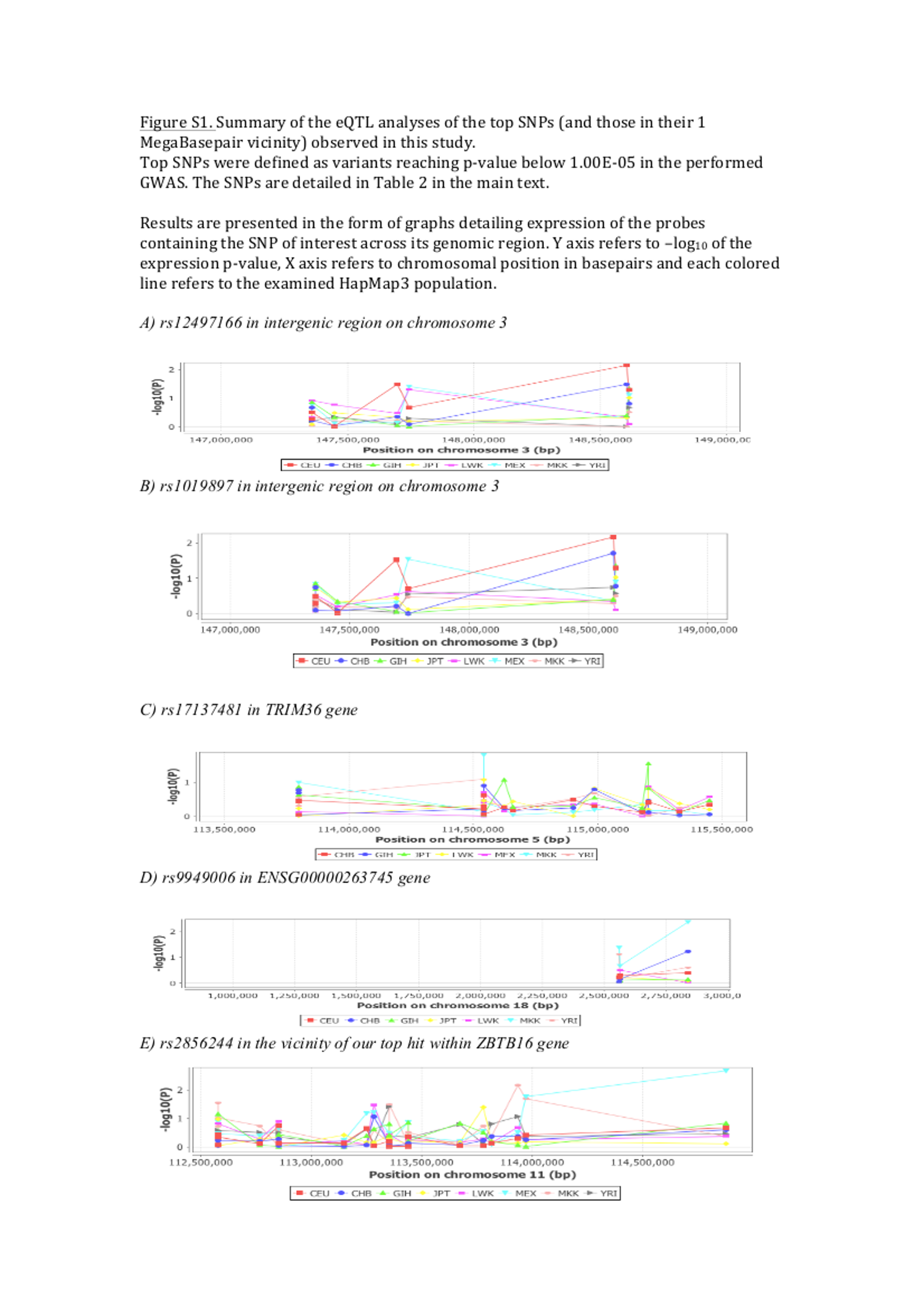

Supplement: S1 Fig — Top SNPs were defined as variants reaching p-value below 1.00E-05 in the performed GWAS. The SNPs are detailed in Table 2 in the main text. Results are presented in the form of graphs detailing expression of the probes containing the SNP of interest across its genomic region. Y axis refers to –log10 of the expression p-value, X axis refers to chromosomal position in basepairs and each colored line refers to the examined HapMap3 population. A) rs12497166 in intergenic region on chromosome 3. B) rs1019897 in intergenic region on chromosome 3. C) rs17137481 in TRIM36 gene. D) rs9949006 in ENSG00000263745 gene. E) rs2856244 in the vicinity of our top hit within ZBTB16 gene. (TIFF) [file pone.0122501.s008.tiff]

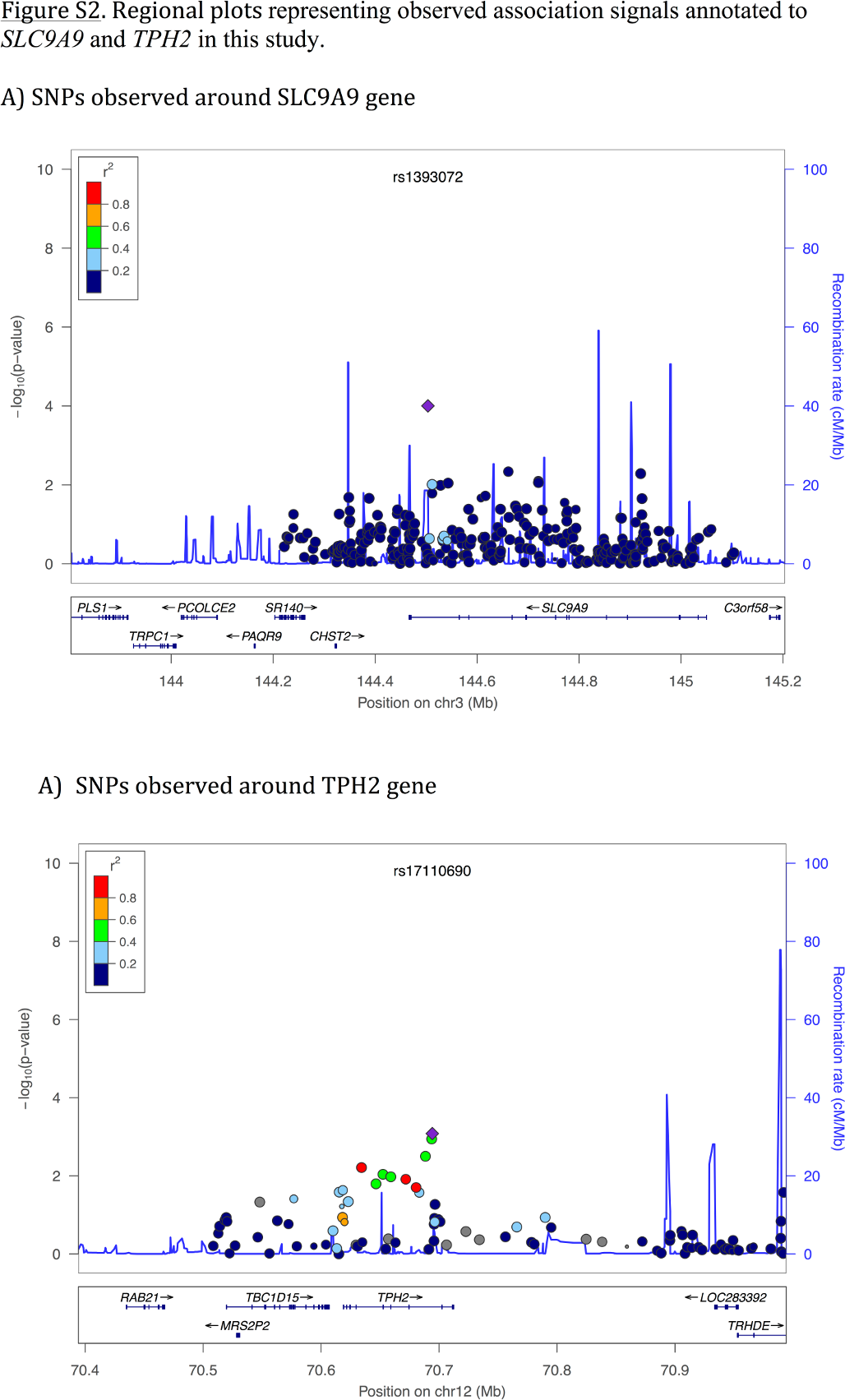

Supplement: S2 Fig — A) SNPs observed around SLC9A9 gene. B) SNPs observed around TPH2 gene (TIFF) [file pone.0122501.s009.tiff]
